# Evolutionary dynamics of the proanthocyanidin biosynthesis gene *LAR*

**DOI:** 10.1186/s12864-025-12429-5

**Published:** 2025-12-19

**Authors:** Maria F. Marin-Recinos, Boas Pucker

**Affiliations:** Plant Biotechnology and Bioinformatics, Institute for Cellular and Molecular Botany - IZMB, University of Bonn, Kirschalle 1, 53115 Bonn, Germany

**Keywords:** Proanthocyanidin, Leucoanthocyanidin reductase, Flavonoid biosynthesis, Gene duplication, Evolution, Subfunctionalization, LAR1, LAR2

## Abstract

**Background:**

Leucoanthocyanidin reductase (LAR) is a key enzyme in proanthocyanidin (PAs) biosynthesis, catalyzing the conversion of leucoanthocyanidins to catechins. While early steps in the flavonoid pathway are broadly conserved across plant lineages, increasing evidence demonstrates lineage-specific evolutionary trajectories and functional diversification in its terminal branches, particularly in the case of LAR. To explore the evolutionary dynamics and functional divergence of LAR genes, we conducted large-scale comparative and phylogenetic analyses across major plant clades.

**Results:**

The phylogenetic analysis revealed multiple independent duplication events and lineage-specific expansions of *LAR* lineages, particularly among dicots and gymnosperms. In dicots, *LAR1* and *LAR2* were differentially retained and diversified, whereas gymnosperm LAR homologs formed early-diverging clades, suggesting an ancient duplication and potential neofunctionalization. Coexpression analyses across species and tissues indicate paralog-specific expression patterns. Sequence analysis identified both conserved and clade-specific protein domains, supporting functional divergence. Promoter analyses showed differences in transcription factor binding site composition between *LAR1* and *LAR2*, pointing to regulatory sub- or neo-functionalization. Lastly, synteny analyses support the potential absence of *LAR* in multiple *Brassicales* genomes.

**Conclusions:**

LAR shows evidence of evolutionary diversification, shaped by both coding and regulatory changes. These patterns of diversification help explain variation in flavonoid profiles in gymnosperms and angiosperms. Understanding the evolutionary dynamics of LAR not only deepens our knowledge of metabolic pathway evolution but also provides insights relevant to the breeding and metabolic engineering of plant traits related to pigmentation, stress resilience, and nutritional quality.

## Introduction

Proanthocyanidins (PAs), also referred to as condensed tannins, constitute a group of polyphenolic compounds within the flavonoid biosynthesis pathway. As secondary metabolites, PAs play an important role in plant defense mechanisms against insect herbivores and microbial pathogens. Their contribution to herbivore deterrence is largely attributed to the astringent and bitter taste they confer to various plant tissues, which reduces palatability and impairs digestibility in insect pests [1–3]. In addition, PAs exhibit antimicrobial activity against pathogens, including bacteria [4–6] and fungi [7]. Historically, PAs have been utilized as tanning agents for leather preservation and in the regulation of different flavor qualities and profiles in beverages such as wine, teas, and fruit juices [8, 9]. Furthermore, their antioxidant properties have been reported for their potential health benefits in both human and ruminant animal consumption [10]. In addition to their role in plant defense, PAs have been extensively studied for their impact on seed coat pigmentation [11, 12], influencing seed dispersal [13] and dormancy mechanisms [14], thereby contributing to plant reproductive efficacy [15, 16].

**Fig. 1:**
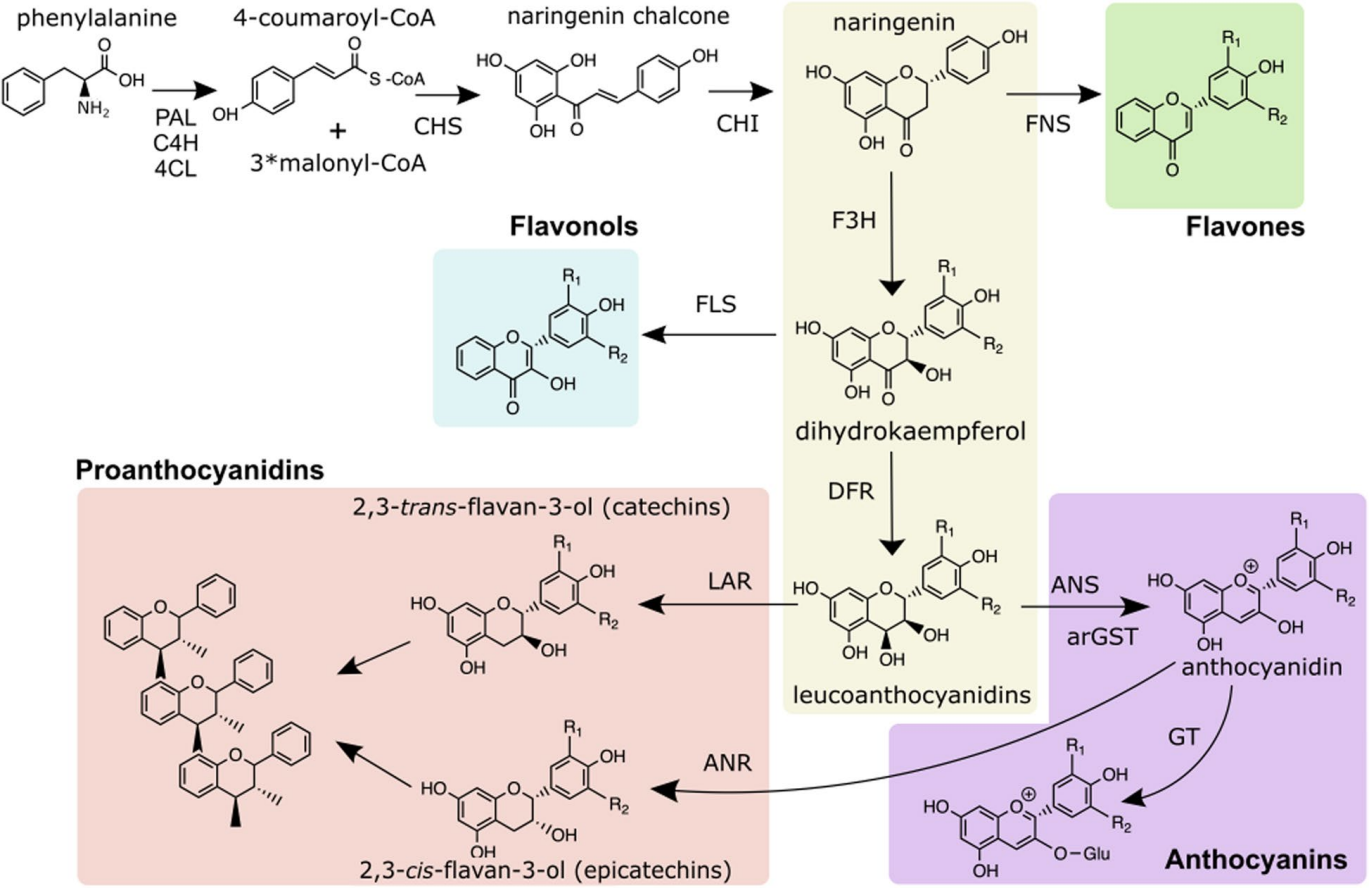
Schematic representation of the general flavonoid biosynthesis pathway. Enzyme names are abbreviated as follows: PAL - phenylalanine ammonia-lyase, C4H - cinnamic acid 4-hydroxylase, 4CL − 4-coumarate-CoA ligase, CHS - chalcone synthase, CHI - chalcone isomerase, FNS - flavone synthase, F3H - flavanone 3-hydroxylase, FLS - flavonol synthase, DFR - dihydroflavonol 4-reductase, ANS - anthocyanidin synthase, arGST – anthocyanin-related glutathione S-transferase, GT - UDP-dependent anthocyanidin- 3-O-glucosyltransferase, ANR - anthocyanidin reductase

PA synthesis represents one branch of the flavonoid biosynthesis pathway, a process extensively elucidated across a wide range of plant species and notably conserved among diverse taxa [17–19]. The synthesis of PAs and anthocyanins, as competing branches of the pathway, share common precursors which involve a series of enzymatic reactions. The key enzymes involved in PA synthesis start with the enzyme dihydroflavonol 4-reductase (DFR) catalyzing the production of leucoanthocyanidins which are then processed by leucoanthocyanidin reductase (LAR) for the conversion into 2,3-*trans*-flavan-3-ols, commonly known as the monomer 2,3-*trans*-(+)-catechin. In a parallel route, anthocyanidin synthase (ANS) converts leucoanthocyanidins to anthocyanidins, which can be reduced by anthocyanidin reductase (ANR) to form 2,3-*cis*-flavan-3-ols, also known as the monomer 2,3-*cis*-(-)-epicatechin. These monomeric units then polymerize to form PA oligomers (Fig. 1).

LAR and ANR play a central role in defining PA composition by synthesizing the flavan-3-ol monomers that act as starter and extension units. Canonically, the primary constituents of these polymers are (+)-catechin, produced by LAR, and (-)-epicatechin, synthesized by ANR. These two monomers serve as the basic building blocks in the formation of proanthocyanidin polymers. However, recent studies in *Medicago truncatula* have reported that the function of LAR extends beyond its traditional role in the synthesis of (+)-catechins [20, 21]. Specifically, LAR has been shown to be responsible for converting 4β-(S-cysteinyl) epicatechin back to epicatechin, a reaction that was previously attributed exclusively to ANR [20, 21]. This dual LAR activity may explain the observed increase in catechin and epicatechin levels under overexpression of *LAR*, indicating that LAR is crucial for regulating both monomer types that serve as starter and extension units in the polymerization process of PAs [20, 21]. Furthermore, Jun et al. demonstrated that leucoanthocyanidin dioxygenase (LDOX) in *M. truncatula* plays a role in the formation of epicatechin starter units from catechin, contrasting with the function of ANS in the conversion of leucocyanidin to cyanidin [21]. Meanwhile, ANR is involved in the synthesis of the extension epicatechin units [21].

In addition to *M. truncatula*, *LAR* genes have been characterized in multiple economically important plants such as *Vitis vinifera* [22], *Theobroma cacao* [23], *Gossypium hirsutum* [24], *Malus domestica* [25], *Pyrus communis* [26], and *Camellia sinensis* [27], all of which belong to a diverse range of plant families and orders. Interestingly, despite the widespread presence of *LAR* genes in many plants, it has been known for years that the model plant *Arabidopsis thaliana* does not harbor a *LAR* gene [19, 28]. However, *A. thaliana* is still capable of synthesizing proanthocyanidins, which provide a dark color to its seed coat [12]. Knowledge obtained from studying the *transparent testa* (*tt*) and *tannin-deficient seed* (*tds*) mutants collection in *A. thaliana* have been essential in understanding the synthesis, regulation, and transport mechanisms involved in the PA production, even in the absence of a functional *LAR* gene [12, 19, 29–32]. The regulation of flavonoid biosynthesis, including the PA branch, is controlled by a well-characterized complex of transcription factors (TFs) known as the MBW complex, consisting of MYB, basic helix-loop-helix (bHLH), and WD40 proteins [33]. In *A. thaliana*, key TFs such as TT2 (R2R3-MYB), TT8 (bHLH), and TTG1 (WD40) regulate the expression of *BANYULS (BAN)* which encodes for ANR involved in the PA production in seeds [12, 34, 35].

Given the widespread occurrence of LAR in various plant lineages and its absence in *A. thaliana*, understanding the evolutionary trajectory of LAR genes can provide valuable insights into their functional diversification and potential compensatory mechanisms in species lacking LAR. Here, we investigate the evolutionary dynamics of *LAR* through a systematic analysis of its presence/absence across land plant lineages. Our findings reveal a deep duplication event at the base of the evolutionary split between gymnosperms and angiosperms that has resulted in many plant species harboring two *LAR* copies, suggesting potential subfunctionalization or neofunctionalization within these paralogs. However, we also identified entire plant lineages without *LAR*. Most notably, *LAR* appears to be largely missing within the *Brassicaceae* family. Additionally, we explored the functional divergence of the two *LAR* lineages in plants with multiple copies, aiming to understand how gene duplication has influenced the evolution and PA biosynthesis.

## Results

### Phylogenetic analysis of leucoanthocyanidin reductase (LAR)

**Fig. 2:**
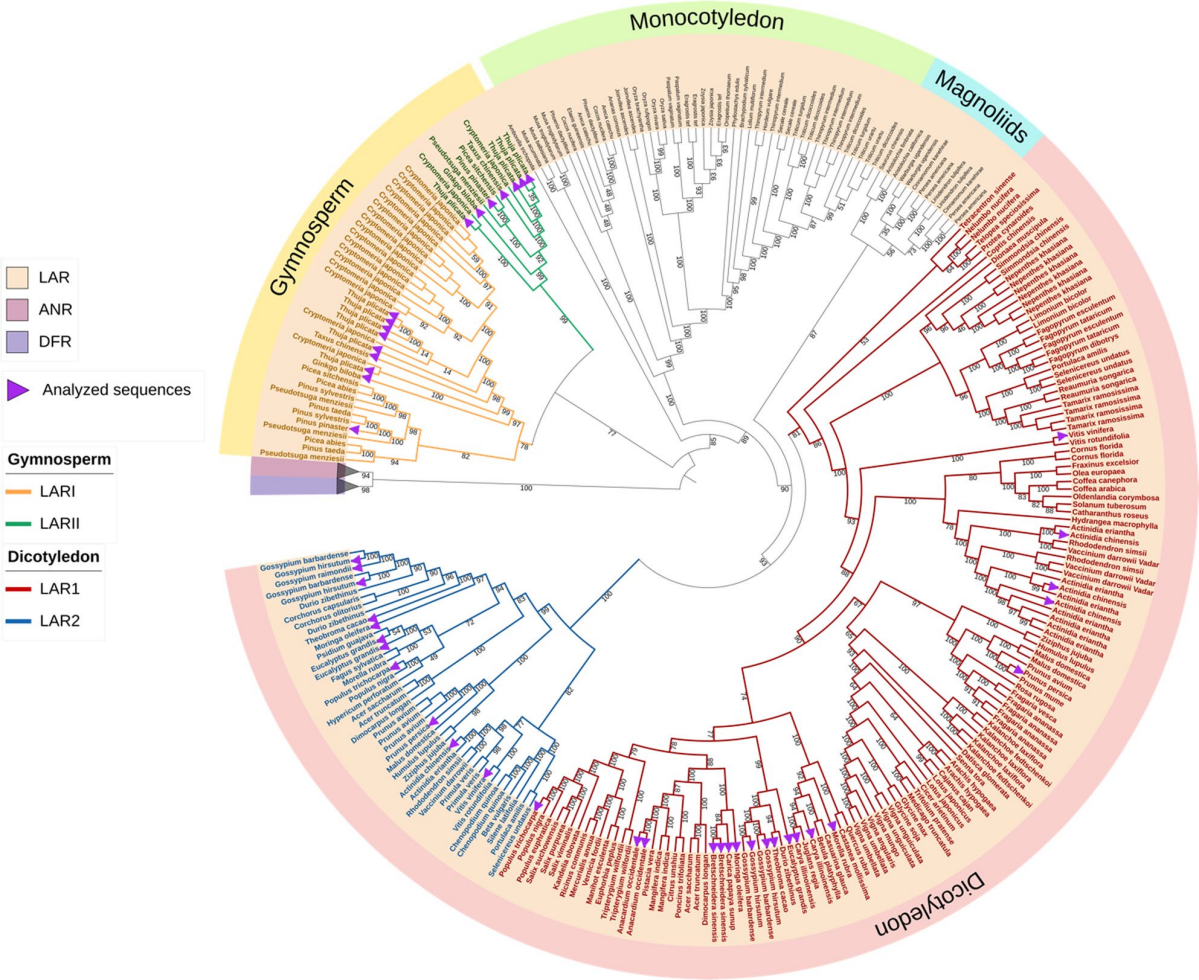
Phylogenetic analysis of LAR, DFR, and ANR sequences in different plant species. Sequences were collected via KIPEs3 results, codon-based alignments were generated with MACSE [36], and the phylogeny was inferred with IQ-TREE2 [37, 38] based on maximum likelihood. Distinct clades of LARI (orange) and LARII (green) in the Gymnosperm group as well as LAR1 (red) and LAR2 (blue) in the Dicotyledon group are highlighted. The purple arrows indicate the sequences used for the synteny and gene expression analysis. The complete tree, including all non-collapsed clades, is available in Additional File 1C [39]

This study explored the phylogenetic relationships of *LAR* genes across a wide range of plant taxa. In total, 287 LAR, 268 DFR, and 112 ANR sequences were retained for alignment and phylogenetic analysis. Four primary clades were identified in the LAR portion of the tree, corresponding to the major plant lineages: gymnosperms, monocotyledons, magnoliids, and dicotyledons (Fig. 2). Within both the gymnosperm and dicotyledon clades, two distinct subclades were identified. These will be referred to here as LARI and LARII for those in the gymnosperm clade (shown in orange and green in Fig. 2, respectively) and LAR1 and LAR2 for those in the dicotyledon clade (shown in red and blue in Fig. 2, respectively). These distinctions are necessary to highlight clear phylogenetic divergence within each lineage and to avoid confusion in downstream analysis.

Interestingly, the presence of separate *LAR* gene copies from the same species in both subclades of gymnosperms (LARI and LARII), as well as in both subclades of dicotyledons (LAR1 and LAR2), implies that an ancient gene duplication event occurred prior to the diversification of each of these groups (Fig. 2). This deep duplication appears to have given rise to two major evolutionary lineages of *LAR* genes in both gymnosperms and dicots. Following this deep duplication, additional lineage-specific duplication events occurred at the family, genus, or species level independently within each clade. For example, in gymnosperms, species such as *Thuja plicata*,* Picea sitchensis*, and *Cryptomeria japonica* present multiple LAR copies distributed across the subclades LARI and LARII. In dicots, the LAR1 subclade represents a particularly large and diverse collection of gene copies. Many species exhibit multiple paralogs within this subclade, pointing to repeated gene duplication events within specific lineages. Families such as *Theaceae*,* Fabaceae*,* Rosaceae*, and *Malvaceae* are especially well represented in LAR1, often with two or more copies per species. The LAR2 subclade, while smaller, also includes multiple gene copies of species present in LAR1. Notably, species such as *Bretschneidera sinensis* (*Akaniaceae*) and *Carica papaya* (*Caricaceae*) both belonging to the *Brassicales* order, contain LAR gene copies only in LAR1, suggesting a potential absence of the LAR2 copy in these lineages. This pattern further supports the idea that, following the initial ancient duplication, LAR1 and LAR2 have undergone independent expansions in different lineages, leading to a potential clade-specific diversification of the *LAR* genes.

### Leucoanthocyanidin reductase duplication event and functional divergence

**Fig. 3:**
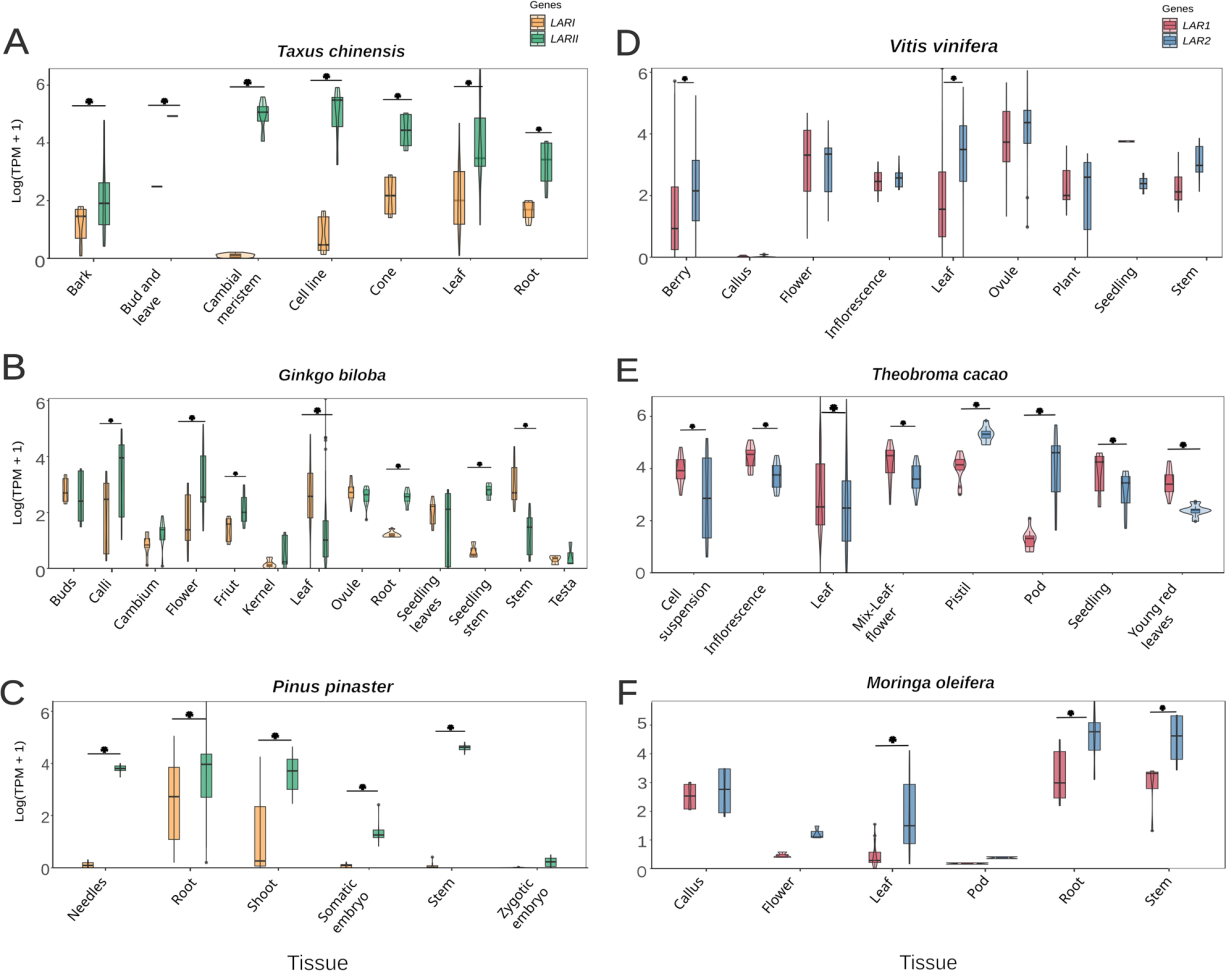
Comparison of the expression of **LARI** (orange) and **LARII** (green) genes in the species **Taxus chinensis** (A), **Ginkgo biloba** (B) and **Pinus pinaster** (C), and **LAR1** (red) and **LAR2** (blue) in the species **Vitis vinifera** (D), **Theobroma cacao** (E), and **Moringa oleifera** (F) genes across different plant tissues. An asterisk above the violin plot indicates a statistically significant difference (p < 0.05) between **LARs** in that specific tissue, based on a linear mixed-effects model followed by estimated marginal means analysis. The complete results of the LMM and EMMs are provided online [39].

**Fig. 4:**
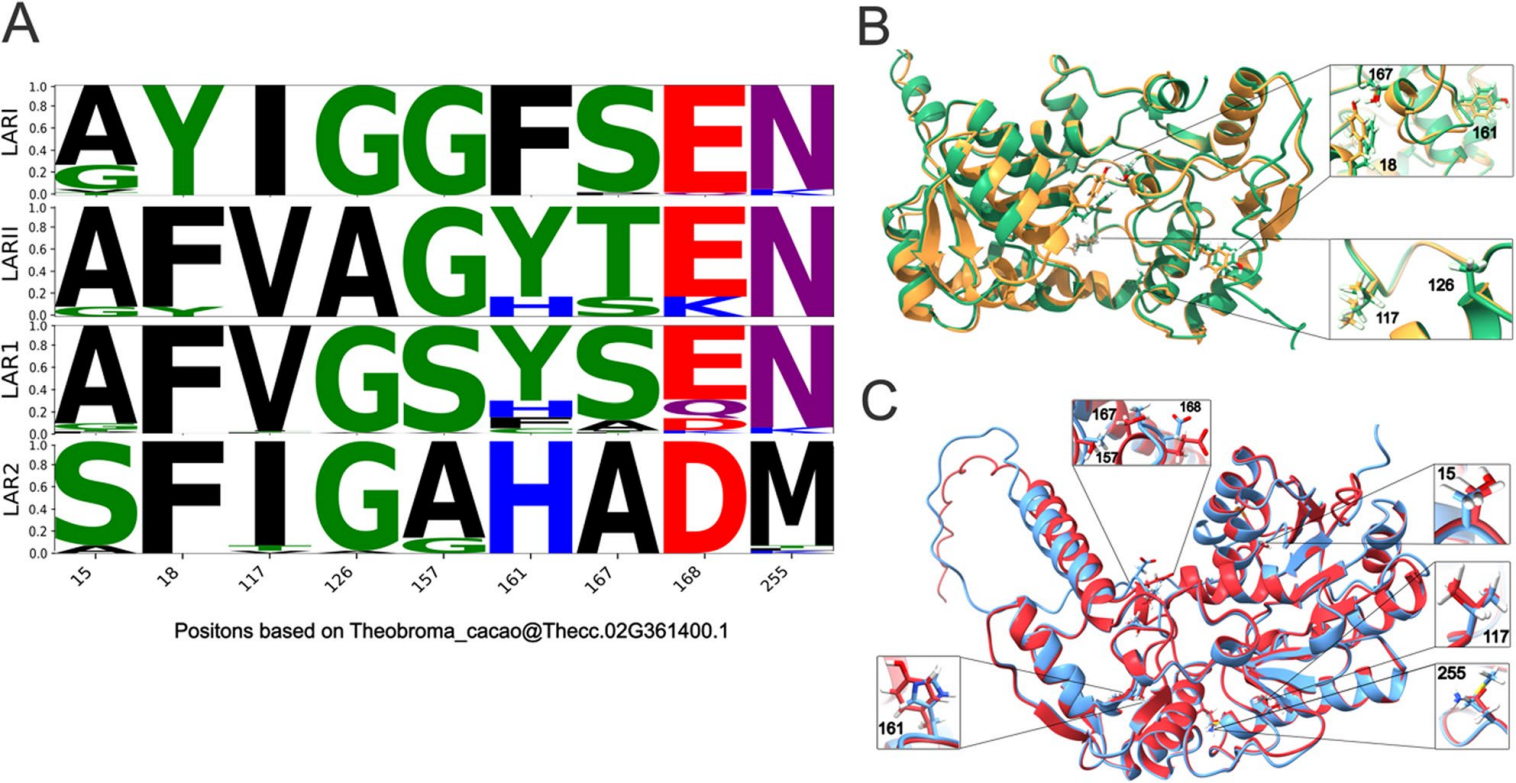
Functional divergence in LAR proteins. **A** Comparative sequence logo showing conservation and variability across LARI and LARII sequences in gymnosperm species, and LAR1 and LAR2 sequences in dicotyledon species. The X-axis represents the position based on *Theobroma cacao* LAR1 (Thecc.02G361400.1) reference sequence, while the Y-axis indicates the frequency in percentage, reflecting sequence conservation at each position. Individual amino acids are color-coded based on their chemical properties: nonpolar/hydrophobic residues (black), polar amino acids with hydroxyl groups (green), polar amino acids with amide residues (purple), positively charged residues (blue), and negatively charged residues (red). The height of each letter within a column corresponds to its relative frequency at that position with taller letters indicating more conserved regions and shorter ones reflecting variability. **B** Structural model of LARI (orange) and LARII (green) of the gymnosperm species *Taxus chinensis*, highlighting five positions with amino acid substitution near the active site of the protein. **C** Structural model of LAR1 (red) and LAR2 (blue) of the dicotyledon species *Theobroma cacao*, highlighting seven positions with amino acid substitution near the active site of the protein

The phylogenetic analysis (Fig. 2) revealed the presence of multiple *LAR* gene copies in several species within both gymnosperms and dicotyledons. This pattern points to an ancestral gene duplication event, followed by divergence within each lineage. Not only these gene copies are phylogenetically distinct, but also, they are not located near each other in the genome (See Additional file 1D [39]). This suggests that they did not arise from recent tandem duplication but rather from older duplication events followed by independent evolutionary paths. Over time, these copies have likely diverged in sequence and regulation, which may reflect differences in function or tissue-specific roles. To explore this potential functional divergence, gene expression patterns of the different LAR paralogs were examined across multiple tissues in representative species presented in the phylogenetic tree. Among gymnosperms, *Taxus chinensis*, *Ginkgo biloba*, *Pinus pinaster*,* Picea sitchensis*, and *Thuja plicata* were analyzed (Fig. 3 [39]), and among dicotyledons the analyzed species included: *Vitis vinifera*, *Theobroma cacao*, *Moringa oleifera*,* Gossypium hirsutum*,* Actinidia chinensis*,* Eucalyptus grandis*,* Populus trichocarpa*,* Carya illinoinensis*, and *Morella rubra* (Fig. 4; [39]). *T. chinensis* showed a significant difference in expression between *TchLARI* (KAH9310580.1) and *TchLARII* (KAH9295439.1). *TchLARII* showed consistently higher expression across all the sampled tissues (Fig. 3A). A similar pattern was observed in *G. biloba*, where *GbiLARII* (GBI00018207) had a significantly higher overall expression than *GbiLARI* (GBI00017466) with tissue-specific expression patterns showing stronger *GbiLARII* expression in seedling stem tissues (p-value = 0.0004) and root tissues (p-value = 0.002), and a stronger *GbiLARI* expression in stem tissues (p-value < 0.0001) (Fig. 3B). In *P. pinaster*, a significant overall expression difference was also detected between *PpiLAR1* (PPI00016665) and *PpiLARII* (PPI00057446), with *PpiLARII* being more highly expressed in needles (p-value < 0.0001), shoots (p-value < 0.0001), and stem tissues (p-value < 0.0001) (Fig. 3C).

In dicots, clear expression differences between paralogs were also identified. In *V. vinifera*,* VviLAR2* (VIT_217s0000g04150.2) showed significantly higher expression than *VviLAR1* (VIT_201s0011g02960.1) in leaf tissues (p-value < 0.0001) and berry tissues (p-value < 0.0001) (Fig. 3D). In *T. cacao*, the gene expression of *TcaLAR1* (Thecc.02G361400.1) was dominant in cells, inflorescence, leaf, seedlings, young red leaves, and mix of leaf and flower tissues while *TcaLAR2* (Thecc.03G028300.1) presented higher expression in pod (p-value < 0.0001) and pistil tissues (p-value < 0.0001) (Fig. 3E). Similarly, in *M. oleifera*, a significant overall expression difference was detected between *MolLAR1* (g8395.t1) and *MolLAR2* (g11921.t1), with *MolLAR2* exhibiting higher expression in roots (p-value = 0.002), leaf (p-value < 0.0001), and stem tissues (p-value = 0.005) (Fig. 3F).

When comparing the 36 sequences of LARI and the 11 sequences of LARII in gymnosperms, both groups showed strong conservation at many positions, numbered according to the *Theobroma cacao* LAR1 (Thecc.02G361400.1) reference sequence (Fig. 4A [39]). Particularly interesting are positions with contrasting differences between LARI and LARII (Fig. 4A). In dicotyledonous species, 106 sequences of LAR1 and 43 sequences of LAR2 were analyzed, revealing comparable conservation with notable divergences at several key residues (Fig. 4A [39]). Conserved regions suggest key functional or structural roles. Amino acid substitutions at five specific positions in gymnosperms (18, 117, 126, 161, 167) and seven in dicots (15, 117, 157, 161, 167, 168, 255) clearly differentiate the gymnosperm LARs (LARI, LARII) from their dicot counterparts (LAR1, LAR2). At position 15, a consistent presence of alanine (A) was found in LARI, LARII, and LAR1, while a substitution to serine (S) was observed in LAR2. At position 18, tyrosine (Y) was exclusive to LARI, while phenylalanine (F) was dominant in LARII and dicots (Fig. 4B). At position 117, valine (V) was retained in both LARII and LAR1, while isoleucine (I) was found in LARI and LAR2. A unique alanine (A) in LARII was observed in position 126, substituting glycine (G) in LARI and dicots. The ICCN motif region centered on position 157 showed a particular divergence in dicots; while serine (S) is highly conserved in LAR1, alanine (A) is more conserved in LAR2. Similar results can be observed at position 167: serine (S) was conserved in LARI and LAR1, while threonine (T) appeared in LARII, and alanine (A) was found in LAR2. Given the proximity of this position to the active site and the polarity differences, this variation was interpreted as functionally relevant (Fig. 4C). An additional substitution at position 168 within a conserved motif replaces glutamate (E) in LARI, LARII, and LAR1 with aspartate (D) in LAR2, reducing the side chain length and potentially altering charge distribution or hydrogen bonding patterns. Finally, position 255, while this has not been reported as part of any LAR-specific motif, showed a predominant conservation of asparagine (N) in all groups except LAR2, which presented a substitution to methionine (M). This polar-to-nonpolar shift, occurring near the active site, was interpreted as likely contributing to a reduction in the catalytic efficiency of LAR2 as reported by the gene expression in Fig. 3E for *T. cacao*. The proximity of several variable positions to conserved motifs and the active site further supports their relevance in the functional diversification of LAR enzymes.

The combined analysis of gene expression patterns and polypeptide seq uence conservation indicates a clear functional differentiation between the *LAR* gene groups. *LAR1* and *LAR2* in dicots, as well as *LARI* and *LARII* in gymnosperms, exhibit distinct tissue-specific expression profiles. These expression patterns are supported at the molecular level by systematic amino acid substitutions revealed through the comparative analysis of LAR sequences. These substitutions differentiate dicots and gymnosperm LARs, as well as within each LAR lineage. Mutations such as Y18F in gymnosperms LARII, and S157A and E168D, both occurring in dicots LAR2, present particular interest due to occurring within conserved motifs.

### Expression analysis among *LAR1 *and *LAR2 *genes

Given the distinct expression patterns and systematic amino acid differences previously identified between LAR1 and LAR2, a coexpression analysis was conducted to further investigate their potential functional divergence and relationships with other metabolic pathways. The results are presented in two tables, in which each *LAR* gene is depicted along with a list of coexpressed genes. For each gene, the Spearman correlation coefficient and gene expression value are provided (See [39]). In *V. vinifera*,* VviLAR1* (VIT_201s0011g02960.1) was notably coexpressed with the genes; VIT_206s0004g08150.1 (*r* = 0.710) encoding trans-cinnamate 4-monooxygenase, a key enzyme in the biosynthesis of flavonoids and lignin, and VIT_213s0067g02870.2 (*r* = 0.657) encoding the enzyme chalcone-flavone isomerase, involved in the flavonoid biosynthesis. In *T. cacao* the analysis identified strong correlations between *TcaLAR1* (Thecc.02G361400.1) and a diverse set of high expressed protein-coding genes involved in stress responses such as proline-rich protein-1 (Thecc.04G253900.1, *r* = 0.666) and the bax inhibitor-1 (Thecc.04G271000.1, *r* = 0.683). Additionally, genes involved in signaling pathways like Calmodulin7 (Thecc.09G265600.2, *r* = 0.763) which plays an important role in seedling development, and vesicle trafficking like ADP-ribosylation factor (Thecc.01G320100.1, *r* = 0.708) and ESCRT-related protein CHMP1B (Thecc.06G106700.1, *r* = 0.695). *MolLAR1* (g8395.t1) in *M. oleifera*, presented strong correlation with genes encoding metallothionein-like protein 1 (g23076.t1, *r* = 0.702), copper transport protein ATX1 (g22635.t1, *r* = 0.701), and Profilin2 (g19932.t1, *r* = 0.682).

Regarding the genes coexpressed with *LAR2*, in *V. vinifera* the gene expression of *VviLAR2* (VIT_217s0000g04150.2) showed stronger correlation with an isoform of the same gene (VIT_217s0000g04150.7, *r* = 0.741), followed by genes encoding sigma intracellular receptor 2 (VIT_212s0059g02290.1, *r* = 0.664), sucrose transport protein (VIT_218s0076g00250.1, *r* = 0.658), and patatin-like protein 2 (VIT_218s0001g10830.1, *r* = 0.651). In *T.cacao*, *TcaLAR2* (Thecc.03G028300.1) coexpressed strongly with the genes encoding the protein SPIRAL1 (Thecc.02G328900.1, *r* = 0.681), GTP-binding nuclear protein (Thecc.09G120500.1, *r* = 0.722), and 60 S ribosomal protein (Thecc.01G108600.1, *r* = 0.707). Lastly, *MolLAR2* (g11921.t1) in *M. oleifera* showed coexpression with genes encoding proteins such as WALLS ARE THIN1 (g3827.t1, *r* = 0.684), Major latex protein-like 43 (g15381.t1, *r* = 0.672), and triose-phosphate/phosphate translocator (g12330.t1, *r* = 0.665).

Coexpression analyses further supported divergence among LAR paralogs, with *TcaLAR1* and *MolLAR1* showing strong positive correlations with genes involved in protein regulation and stress response. Likewise, the coexpression profile of *TcaLAR2* was strongly related to tissue-specific expression, specifically to reproductive development, consistent with its higher expression in pods and flower tissues.

### Regulatory divergence between *LAR1 *and *LAR2 *gene promoters and identification of transposable elements

To explore the regulatory divergence between the two *LAR* gene copies, promoter sequences from *V. vinifera*,* T. cacao*, and *M. oleifera* were extracted and analyzed for transcription factor binding motifs and transposable elements (TE) insertions. This species-specific comparison provides additional insights into the functional differentiation of these LAR paralogs and highlights the potential influence of TEs on *LAR* gene regulation, particularly in the context of proanthocyanidin biosynthesis.

**Fig. 5:**
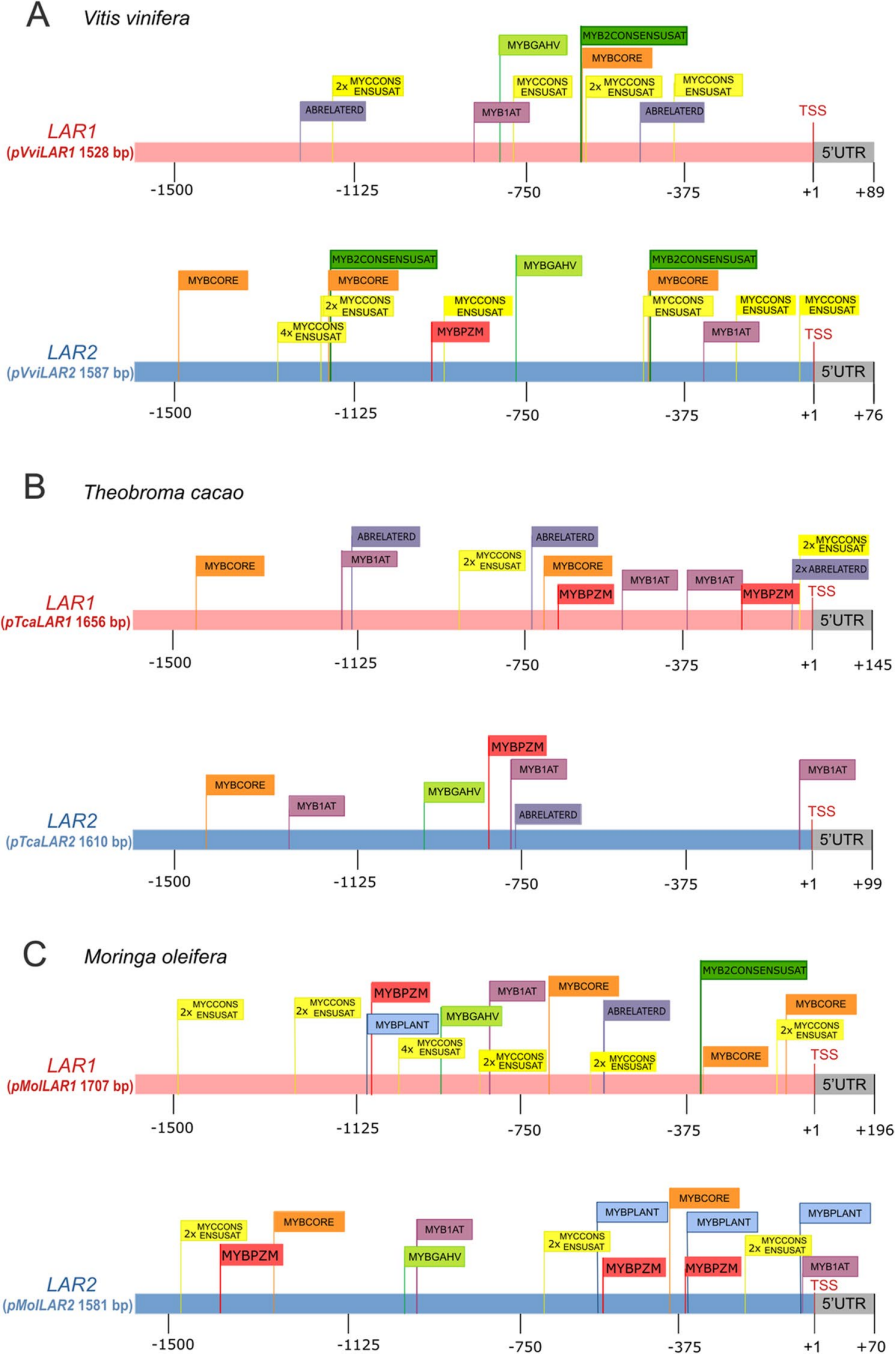
Promoter region constructs with putative cis-acting regulatory elements (CREs) predicted by the PLACE database [40]. **A** *pVviLAR1* and *pVviLAR2 *in* Vitis vinifera. ***B** *pTcaLAR1* and *pTcaLAR2* in *Theobroma cacao*. **C** *pMolLAR1* and *pMolLAR2 *in* Moringa oleifera.* CREs are displayed above each promoter schematic, and transcription start sites (TSS) are indicated in red. The 5’ untranslated region (5’ UTR), shown in gray, is a schematic representation and not proportional to bp length. A comprehensive list of CREs for each promoter is available online [39].

In *V. vinifera*, both promoters *pVviLAR1* and *pVviLAR2* included motifs associated with flavonoid biosynthesis gene regulation, notably those bound by MYB transcription factors. *pVviLAR1* exclusively contained ABRELATERD1 motifs and shared with *pVviLAR2* the presence of MYCCONSENSUSAT, MYBCORE, MYB1AT, MYBGAHV, and MYB2CONSENSUSAT. In contrast, *pVviLAR2* uniquely harbored a MYBPZM motif. While both genes showed considerable overlap in motif composition, *pVviLAR2* presented elements in closer proximity to the transcription start site (TSS) and a more varied motif composition, which may reflect a broader regulatory role (Fig. 5A). Additionally, TEs annotated with EDTA revealed a Helitron TE insertion in *pVviLAR1* and a Mutator-like TIR transposon in *pVviLAR2.*

In *T. cacao*, both promoter regions displayed a diverse motif landscape sharing elements such as MYB1AT, MYBCORE, MYBPZM, and ABRELATERD1. Notably, *pTcaLAR1* presented a higher number of motifs overall and uniquely included multiple MYCCONSENSUSAT elements, while *pTcaLAR2* was characterized by the presence of MYBGAH (Fig. 5B). In *M. oleifera*, the promoter region *pMolLAR1* presented a richer and more dense motif profile compared to *pMolLAR2*. Shared motifs included MYB1AT, MYBCORE, MYCCONSENSUSAT, MYBPZM, and MYBPLANT. In addition, ABRELATERD1 and MYB2CONSENSUSAT were exclusively found in *pMolLAR1.* (Fig. 5C). No TE insertions were detected in the promoter regions of either *LAR* gene in *T. cacao* and *M. oleifera*. Collectively, these findings reveal both conservation and divergence in the regulatory landscapes of *LAR1* and *LAR2* across species. MYB and MYC motifs were consistently found and are likely essential for proanthocyanidin biosynthesis regulation. However, the presence of TEs in the *LAR* promoters of *V. vinifera*, in contrast with their absence in *T. cacao* and *M. oleifera*, point toward species-specific regulatory adaptations, supporting the hypothesis that promoter evolution via motif variation and TE insertions may have contributed to species-specific expression patterns of LAR paralogs.

### Loss of LAR in families *Cleomaceae* and *Brassicaceae*

**Fig. 6:**
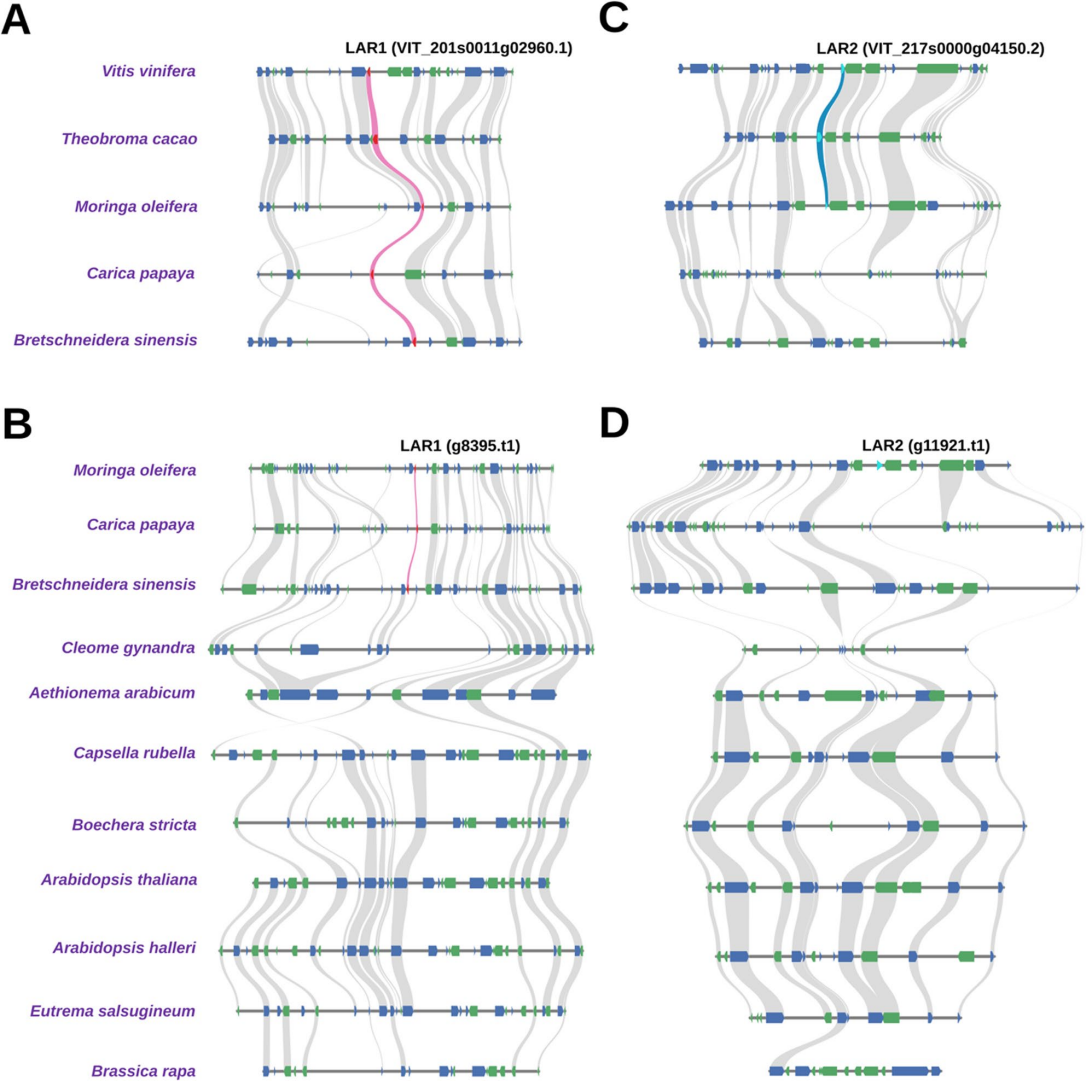
Synteny gene identification of species in the *Brassicales* order. **A** Synteny around *LAR1* (indicated in pink) among the genome sequences of *V. vinifera*,* T. cacao*,* M. oleifera*,* C. papaya*, and *B. sinensis.* **B** Synteny around *LAR1* among the genome sequences of *M. oleifera*,* C. papaya*,* B. sinensis*,* C. gynandra*,* A. arabicum*,* C. rubella*,* B. stricta*,* A. thaliana*,* A. halleri*,* E. salsugineum*, and *B. rapa.* **C** Synteny around *LAR2* (indicated in light blue) among the genome sequences of *V. vinifera*,* T. cacao*,* M. oleifera*,* C. papaya*, and *B. sinensis.* **D** Synteny around *LAR2* among the genome sequences of *M. oleifera*,* C. papaya*,* B. sinensis*,* C. gynandra*,* A. arabicum*,* C. rubella*,* B. stricta*,* A. thaliana*,* A. halleri*,* E. salsugineum*, and *B. rapa.* A complete list of syntenic gene positions and coordinates used for this analysis is provided in [39]

To confirm the absence of LAR in the *Cleomaceae* and *Brassicaceae* families we performed a synteny analysis that first compared the genome sequences of three species belonging to different *Brassicales* families: *Moringa oleifera* (*Moringaceae*), *Carica papaya* (*Caricaceae*), and *Bretschneidera sinensis* (*Akaniaceae*) in context with the outgroup species *Theobroma cacao* (*Malvaceae*) and *Vitis vinifera* (*Vitaceae*) (Fig. 6A). Microsynteny confirmed the presence of *LAR1* among above-mentioned species of the *Brassicales* order and the outgroups. However, when repeating the analysis only among members of the *Brassicales* order, this time including *Cleome gynandra* (family *Cleomaceae*), a close relative of the *Brassicaceae*, as well as the *Brassicaceae* species: *Aethionema arabicum*,* Capsella rubella*,* Boechera stricta*,* Arabidopsis thaliana*,* Arabidopsis halleri*,* Eutrema salsugineum*, and *Brassica rapa*, no *LAR1* gene was detected among the *Cleomaceae* and *Brassicaceae* species (Fig. 6B).

After discovering two independent LAR clades through the phylogenetic analysis, it was necessary to determine whether *LAR2* is present among the *Brassicales* families. This synteny analysis included as reference the genome sequence of *V. vinifera* with the region where *LAR2* is localized. *LAR2* was only observed in the syntenic position between *V. vinifera*, *T. cacao*, and *M. oleifera*, corresponding to the *LAR2* presence in these species as revealed by the phylogenetic analysis (Fig. 6C). Like *LAR1*, *LAR2* was also not detected in multiple *Brassicales* families in this synteny analysis (Fig. 6D).

## Discussion

The flavonoid biosynthesis pathway is generally believed to be well conserved across different plant taxa [18, 41]. The evolution of flavonoids has been extensively studied, including the distinct evolutionary mechanisms that led to some of the individual branch products of the pathway [42–44]. Evolution of this biosynthetic pathway and novel traits are often achieved by changes in transcription factors [45–47]. Such findings have provided significant insights into the genetic and enzymatic variations that contribute to the diversity of flavonoid compounds among plant species [48–50]. Research has revealed that gene duplications and subsequent functional divergences are key processes in the evolution of flavonoid biosynthesis [45, 51]. These genetic variations result in a wide array of flavonoid structures, many of which have well-reported biological functions, such as antioxidant activity [52], UV protection [53], and attraction of pollinators and seed dispersers [54]. Additionally, studies have highlighted the role of regulatory genes in controlling the expression of genes encoding enzymes involved in flavonoid production [3, 47, 55]. Our findings on LAR highlight the mechanism by which gene duplication, divergence, and loss of the *LAR* gene have driven functional diversification in the context of proanthocyanidin biosynthesis across plant species.

### Evolutionary Dynamics of *LAR* Gene Duplications

The phylogenetic analysis revealed that LAR has undergone complex evolutionary trajectories in both gymnosperms and dicotyledons. Within the dicotyledon group, *LAR* genes are segregated into two major clades, referred to as LAR1 and LAR2, which likely originated from an ancient duplication event in the common ancestor of the eudicotyledons. This bifurcation of *LAR* genes into distinct evolutionary paths denotes selective pressure favoring the maintenance of both paralogs, possibly due to their acquisition of distinct or complementary functions.

Evidence of this phylogeny pattern in LAR has been previously reported by Wang et al. [27], who reported that plant LARs present three distinctive groups, including monocotyledons, gymnosperms, and dicotyledons, with the latter further splitting into two clades. The authors also noted the presence of sequence-level divergence due to a DNA transversion and transition in a codon of the LAR-specific motif *ICCN* [27]. This finding was corroborated by our results, which also contain a serine residue in *LAR1* substituted by alanine in *LAR2.* However, we also observed a similar duplication pattern in gymnosperms, with gene copies clustering into two distinct clades, LARI and LARII. Similar to dicots, these clades do not correspond to recent tandem duplication, as the copies are found in separate genomic regions, suggesting an ancient duplication event followed by long-term divergence. Notably, LARII in gymnosperms shows strong amino acid sequence similarity to the LAR paralogs in dicots, supporting the hypothesis that the duplication originated in a common ancestor of seed plants [56]. Under this scenario, one of the ancestral copies was potentially retained and further duplicated within dicots, giving rise to LAR1 and LAR2, while no such duplication has been observed in monocots. This evolutionary model implies that the *LAR* gene family underwent differential retention and lineage-specific diversification across major plant groups. Additionally, *LARII* in gymnosperms tend to show higher expression across several tissues compared to *LARI*, inferring the possibility that regulatory divergence may also have contributed to their functional differentiation and evolutionary paths.

### Functional divergence between LAR1 and LAR2

The functional differentiation of LAR1 and LAR2 is supported not only by their phylogenetic divergence but also by their distinct expression profiles. Gene expression analyses across several dicot species revealed consistent differences between the two copies. For instance, *TcaLAR1* is predominantly expressed in vegetative tissues such as leaves and seedlings, highlighting its role in primary metabolism and general stress responses. This is further supported by coexpression of *TcaLAR1* with stress-related genes, including *proline-rich protein-1*, *bax inhibitor-1* and *Calmodulin7*, which are known to be involved in abiotic stress tolerance [57–59]. In contrast, *TcaLAR2* presented higher expression in reproductive tissues, such as pods and pistils, indicating a more specialized function in these organs. *TcaLAR2* was found to be coexpressed with genes involved in structural and regulatory processes, including the protein SPIRAL1, known for its expression in tissues undergoing rapid cell expansion [60], these findings support its potential role in reproductive tissue development. This pattern of tissue-specific functional differentiation points to a potential adaptive significance of maintaining multiple *LAR* copies within a given species. This divergence is also evident in gymnosperms. For example, in *T. chinensis* and *G. biloba*, *LARII* consistently showed higher expression across several tissues compared to *LARI*. In *P. pinaster*, *LARII* was strongly expressed in needles and shoots, reinforcing the notion that regulatory divergence is an independent outcome following LAR duplication in gymnosperms and dicots. Variation in expression patterns among species suggests that these LAR duplicates may have undergone lineage-specific adaptations shaped by ecological or developmental constraints. These findings align with previous studies that have demonstrated how gene duplication events can give rise to paralogs through subfunctionalization or neofunctionalization allowing species to optimize their resources and improve their environmental adaptation [61, 62].

Sequence comparisons among LAR paralogs across gymnosperms and dicots further revealed both conserved patterns and lineage-specific divergences, particularly at functionally relevant motifs. Among gymnosperm copies, LARII exhibited sequence similarity to dicot LARs (LAR1 and LAR2), especially at conserved residues such as positions 157 and 168. This pattern, along with the consistently higher expression levels of *LARII* compared to *LARI*, supports the hypothesis that LARII may have retained more ancestral structural features, making it a more functionally conserved paralog than LARI. Additionally, the sequence variations identified between LAR1 and LAR2 reinforce the evidence for their functional divergence. One of the most prominent mutations involves S157A within the conserved *ICCN* motif, a region implicated in substrate binding and catalytic specificity of LAR enzymes [27, 63]. Polar amino acid residues like serine are known to facilitate hydrogen bonding with substrates or cofactors, supporting catalytic activity and proper substrate binding [64, 65]. In contrast, nonpolar residues such as alanine contribute primarily to structural stability and maintaining the integrity of the protein through hydrophobic interactions. The substitutions S157A and S167A in LAR2 may therefore alter the enzyme’s interaction dynamics or catalytic behavior. Although changes in protein structure do not directly control gene expression, structural divergence can result in functional specialization, which may in turn be accompanied by differential expression in different tissues. In this context, the specialized expression of *TcaLAR2* in reproductive tissues could reflect a shift in its biochemical role, favored by changes in substrate preferences, activity, or protein-protein interactions. Supporting this, Liu et al. [23] reported a similar expression profile of a *T. cacao LAR* gene located on chromosome 3, which matches the position of *TcaLAR2* in this study. Liu et al. reported a higher expression in pods and seeds, supporting the idea that *TcaLAR2* may be functionally specialized for reproductive tissue processes such as proanthocyanidin biosynthesis during pod development [23]. Another substitution potentially linked to tissue-specific expression is E168D in LAR2, a variation that also lies within a conserved motif. Although both glutamate and aspartate are negatively charged, the shorter side chain of aspartate can reduce enzyme efficacy and alter substrate specificity. While these effects have not been specifically demonstrated in LAR proteins, similar glutamate-aspartate substitutions in other enzymes have been shown to impair catalytic activity and binding properties [66–68]. In addition, the substitution N255M, which lies adjacent to the active site, was also identified. Asparagine (N), conserved in LARI, LARII, and LAR1, is replaced by methionine (M) in LAR2. As a polar residue, N255 commonly participates in hydrogen bonding, these bonds help stabilize the configuration of the active site [69]. Additionally, the presence of a polar sidechain can contribute to the potential electrostatic interactions, enhancing substrate binding [70]. Contrarily, M255 in LAR2 is larger and has a nonpolar sidechain, which may disrupt these interactions with a possible decrease in catalytic efficiency through altered substrate positioning or transition state stabilization [71, 72]. The proximity of these changes to conserved motifs and active sites suggests that they may impact enzyme stability as well. These findings align with a previous report [27], which highlighted the importance of specific amino acid changes in the evolution of LAR in *C. sinensis*.

Together, these amino acid substitutions in LAR sequences point to biochemical divergence that may contribute to tissue-specific functions. Their location within or in proximity to conserved motifs can be interpreted as indicative of evolutionary shifts likely affecting substrate affinity or motif function. While our findings provide evidence for structural differences, they also propose new directions for understanding how such molecular changes might relate to the distinct functional roles observed in LAR across species.

### Role of CREs and TEs in divergence of LAR1 and LAR2 regulation

The diversification of the flavonoid biosynthesis pathway in angiosperms is closely tied to the evolutionary plasticity of its regulatory hotspots [48, 73], including those involved in the reduction of leucoanthocyanidins to catechins via LAR [74, 75]. In this study, the promoter regions of *LAR1* and *LAR2* from *V. vinifera*,* T. cacao*, and *M. oleifera* were analyzed to assess regulatory divergence through the identification of cis-regulatory motifs (CREs) and transposable elements (TEs). Our findings suggest that promoter divergence, modulated by cis-element variation and TE insertions, may explain functional specialization of the two **LAR** paralogs in a lineage-specific manner. Promoter region analysis revealed a common pattern involving MYB, and bHLH binding elements across all species, consistent with the well-documented role of members of these TFs families in regulating flavonoid biosynthesis [76]. Several MYB TFs are established regulators of the phenylpropanoid and flavonoid pathways [77, 78], specific members such as MYBPA1 and MYBPA2 in *V. vinifera*, and MYB5 and MYB14 in *M. truncatula* have been shown to activate the expression of *LAR* and *ANR* genes, thereby controlling proanthocyanidin biosynthesis [79–81]. Similarly, bHLH transcription factors, which function as part of the MBW complex, not only assist MYBs in regulating anthocyanin and proanthocyanidin production but also modulate gene expression dynamically in response to developmental stages and environmental stimuli [82]. In this study, the *VviLAR2* promoter presented a higher abundance of MYC-related cis-elements (E-boxes), which are canonical binding sites for bHLH transcription factors such as MYC1, MYC2, and MYCA1 [83–85]. Notably, MYC1 has been shown to interact with R2R3-MYB proteins to coregulate anthocyanins and PA biosynthesis during berry development [84]. The enrichment of bHLH-binding motifs in the *VviLAR2* promoter may therefore contribute to tissue-specific expression of *VviLAR2*, with particularly high levels in grape berries. Additionally, previous studies of the promoter regions of both **LAR** genes in *V. vinifera* have shown that *VviLAR1* expression is sensitive to light conditions, with higher expression under light and repression in the dark, correlating with the activity of bHLH transcription factors such as MYC2 and MYCA1, the latter being upregulated in callus tissues under dark conditions [86]. Our promoter analysis supports these regulatory patterns; *pVviLAR1* presents multiple light-responsive elements (LREs), such as GT1-related motifs [39]. While the presence of MYB and bHLH binding sites in **LAR** promoters does not provide functional evidence, it suggests the potential regulation by these TF families. This supports the hypothesis that *LAR* expression, and by extension, PA biosynthesis may be modulated in response to developmental and environmental cues, consistent with the dual roles of these specialized metabolites in pigmentation and protection [87]. Overall, these findings suggest that *VviLAR1* is predominantly regulated by light-responsive pathways, whereas the enrichment of MYC-binding motifs in *pVviLAR2* implies that this paralog may be more responsive to bHLH-mediated regulation.

In *T. cacao*,* pTcaLAR1* exhibited a higher overall abundance of MYB motifs. Among these elements is MYBCORE (CNGTTR), a canonical target for R2R3-MYB proteins. This motif is recognized by *MYB.Ph3* in *Petunia hybrida*, known to be involved in regulating anthocyanins and PA biosynthetic genes in petal epidermal cells [88]. Moreover, *pTcaLAR2* contains the MYBGAHV motif (TAACAAA) which is not found in *pTcaLAR1*. This element is the specific binding site for GAMYB, a gibberellin (GA) inducible MYB transcription factor, shown to activate GA-responsive genes such as high-pl α-amylase in barley aleurone cells [89]. The presence of this cis-element in *pTcaLAR2* suggests that this paralog may be selectively responsive to GA signaling, potentially linking its regulation to flower and seed development. GA is known to play critical roles in the transition from vegetative to reproductive phase including the formation of floral organs such as seed and pollen tube growth [90, 91]. This aligns with the enrichment of POLLEN1LELAT52 motif in *pTcaLAR2*, a cis-element known for pollen-specific expression [92]. These findings further support the hypothesis that *TcaLAR2* may be subject to a more restricted regulatory profile, with activity concentrated mostly around reproductive organs. In *M. oleifera*, *pMolLAR2* is characterized by a higher number of flavonoid-related motifs, including MYBPZM and MYBPLANT with the latter being reported as binding site for transcription factors such as *AmMYB305* and *AmMYB340* in *Antirrhinum majus*, which regulate phenylpropanoid and lignin biosynthesis [93].

The differential presence of TE insertions within the **LAR** promoter regions may also play a significant role in the regulatory divergence between *LAR1* and *LAR2*. In *V. vinifera*, both promoters contain TE insertions, but differ in their composition and position. These elements are inserted in different regions within the promoters and genes, likely influencing their expression. TE insertion can influence gene regulation by introducing or removing cis-regulatory elements, modulating chromatin accessibility, or even acting as alternative enhancers or repressors [94]. For instance, LTR retrotransposons have been associated with pigmentation loss in crops such as grape, blood orange, and apple by modulating the expression of anthocyanin biosynthesis genes [95–97]. However, the extent to which these regulatory disruptions in the anthocyanin pathway influence proanthocyanidin production remains unclear. By contrast, no transposable elements were detected in the *LAR* promoters of *T. cacao* and *M. oleifera*, indicating a more conserved promoter architecture.

Across all three species, promoter region analyses revealed the presence of MYB and bHLH binding motifs associated with LAR regulation, suggesting that particular members of these TF families contribute to the conserved control of PA biosynthesis. However, the distribution and abundance of these conserved motifs, along with their proximity to the TSS and presence of species-specific or paralog-specific elements, supports a model in which **LAR **gene duplicates have acquired distinct regulatory profiles.

### Insights into the loss of **LAR** in the families *Cleomaceae* and *Brassicaceae*

The absence of *LAR* genes in the *Cleomaceae* and *Brassicaceae* families, as revealed by the synteny analysis, presents an interesting case of metabolic adaptation. While previous studies have not reported the presence of *LAR* homologs in *A. thaliana* [20] or in species of the *Brassica* genus [98], our results extend this observation to a broader sampling within the family and beyond. Given that *A. thaliana* predominantly accumulates PAs composed of (-)-epicatechin, the presence of (+)-catechins, and consequently a functional *LAR* gene, has not yet been reported in this species [20]. The retention of other proanthocyanidin biosynthesis genes in *Brassicaceae* [98] points to a functional compensation mechanism or a shift in metabolic compounds that led to LAR not being necessary in the production of proanthocyanidins. It is possible that the pathway in *Brassicaceae* species has evolved to rely solely on ANR activity for the synthesis of PAs, unlike *M. truncatula*, which dual activity of LAR has been reported in the production of catechins and its involvement with ANR in the synthesis of epicatechins [21]. This selective loss within the common ancestor of the *Cleomaceae* and *Brassicaceae* families hints at the likelihood that alternative enzymatic pathways or redundant mechanisms might compensate for the absence of LAR. Similar patterns of **LAR** loss have been observed in other lineages. For example, both *Zea mays* and *Sorghum bicolor* lack apparent *LAR* homologs [99]. While maize shows low PA levels and relies solely on ANR to produce epicatechin-based PAs, sorghum is a PA-rich species with a more complete set of regulatory transcription factors, including TT2-type MYBs, synthesizing catechin and procyanidin B3 dimers as major PA components [99]. This divergence illustrates that although both species share the absence of LAR, their metabolic compensation strategies and PA profiles are lineage-specific. Such metabolic shifts are not uncommon in plant specialized metabolism. Similar cases include the mutual exclusivity of anthocyanin and betalain pathways in the *Caryophyllales* order [100, 101], or the presence of glucosinolates that are largely confined to *Brassicales* [102]. These examples highlight how evolutionary pressures shape biosynthetic pathways differently across taxa.

Although *A. thaliana* has been instrumental in elucidating the core steps of the flavonoid biosynthesis, its limitations, such as the lack of LAR, highlight the importance of exploring additional model species. Recent reviews have proposed new model systems better suited for studying the full diversity of PA biosynthetic pathway, particularly in species where both LAR and ANR are present [31, 103]. In this context, crops such as *V. vinifera* and *T. cacao*, where the LAR function is well-documented and linked to seed development and PA accumulation [22, 23], could provide opportunities to better understand species-specific enzymatic adaptations.

This study confirms and adds to previous findings by providing additional evidence that LAR is likely absent from a broader range of families within the *Brassicales* order. The release of new genome sequences from additional *Brassicales* species in the next years will allow to narrow down these loss events, which might affect more families beyond *Cleomaceae* and *Brassicaceae*. Together, these findings highlight the dynamic nature of plant specialized metabolism emphasizing the value of integrating model and non-model systems to fully grasp the evolution and diversification of biosynthetic pathways.

## Conclusion

We conclude that the four distinct *LAR* clades have functional differences i.e., with respect to gene expression and enzymatic properties. However, a universal pattern of functional difference was not discovered and might not exist due to secondary evolutionary events. Interestingly, LAR1 was represented by a higher number of sequences compared to LAR2. While this may partly reflect differences in species-richness, genome sequence availability, and annotation quality, it could also indicate that LAR1 is more broadly retained and functionally important across dicots. This overrepresentation of LAR1 may reflect a central role in proanthocyanidin biosynthesis and stress responses as indicated by coexpression results. Future research could focus on exploring the biochemical properties of LAR1 and LAR2 enzymes in greater detail to better understand whether these differences result from subfunctionalization or neofunctionalization. Additionally, investigating the interaction dynamics influencing substrate specificity could provide valuable insights into the evolutionary and ecological significance of proanthocyanidin metabolism in plants. Most importantly, future studies are encouraged to differentiate between members of the LAR1 and LAR2 clade in dicots or LARI and LARII clade in gymnosperms, respectively, when reporting about LAR.

## Methods

### Data collection

Two primary types of sequence datasets were collected for this study: genome assemblies and corresponding annotations for synteny analysis, and *LAR*, *ANR*, and *DFR* coding and peptide sequences for phylogenetic construction.

#### Genomic data for synteny analysis

The genome assemblies and corresponding gene annotation of 11 species were retrieved from Phytozome and NCBI genome database (Table 1). These data were used for synteny analysis to evaluate genomic neighborhood conservation of the *LAR* gene and support putative loss events (See methods Sect. Synteny analysis).

#### Candidate sequences for phylogenetic analysis

To reconstruct the evolutionary history of *LAR*, *DFR*, and *ANR* genes, candidate orthologs were identified across a broad range of plant species using KIPEs v3 with default parameters [104]. Protein databases were screened using the bait and reference data FlavonoidBioSynBaits_v3.3 provided by KIPEs3, and only hits showing a 100% match in the conserved amino acid residues were retained to infer presence in a plant lineage. Partial candidates were inspected when inferring absence of LAR. The resulting sequences were then cleaned and mapped to their corresponding coding sequences (CDS) using a custom pipeline (see methods Sect. Alignment and phylogenetic tree construction). The species tree used to guide synteny comparisons and phylogenetic interpretation was obtained from [105].

### Gene annotation

Since no annotation of the coding sequences was publicly available for *Moringa oleifera*, BRAKER v3.0.8 [106] with default settings was applied to generate a structural annotation of protein-encoding genes. To support the annotation process with hints, several RNA-seq data sets were retrieved from the Sequence Read Archive [107, 108] and aligned to the genome sequence with HISAT v2.2.1 [109] using default parameters. Samtools v1.20 [110] was used to sort the resulting BAM file which was then provided to BRAKER3 [106].

### Alignment and phylogenetic tree construction

A collection of LAR, DFR, and ANR sequences were identified using KIPEs v3 [111]. Sequences were only considered if a strict match of 100% was found in the set of conserved amino acid residues defined in the KIPEs3 bait file, which ensures a high-confidence candidate identification. These sequences were selected for further analysis. To improve tree readability, sequences labeled as “putative”, “predicted”, or repetitive sequences were removed using a custom python script (clean_fasta.py), which is publicly available on GitHub: https://github.com/mariamarinr/LAR_Evolution/. This filtering step was designed to preserve taxonomic representation, retaining at least one high-confidence LAR sequence per major plant lineage. After the sequence cleaning, the corresponding coding sequence (CDS) was retrieved for each polypeptide sequence to enable codon-aware alignments. This was done using a custom pipeline that cross-referenced gene identifiers from the amino acid FASTA headers with available genome sequence annotations and CDS files (extract_kipes_cds.py) on GitHub: https://github.com/mariamarinr/LAR_Evolution/. Only sequences with matching identifiers to their corresponding polypeptide sequences were retained. In cases where formatting differences in the headers occurred between CDS and polypeptide sequence files, identifiers were standardized to ensure a perfect match. This ensured consistency between protein-level and nucleotide-level data for downstream phylogenetic analysis (see [39] for a complete list). Codon-aware multiple sequence alignments were constructed using MACSE v2.07 [36, 112] with default parameters. An initial phylogenetic tree was constructed with FastTree v2.1.10 [113] using the WAG substitution model and without bootstrap support for rapid visualization. To confirm the phylogenetic structure and arranged relationships, a more robust analysis was performed using IQ-TREE v2.0.7 [37, 38], with ultrafast bootstrap and 1000 replicates. The best-fit substitution model was identified using IQ-TREE’s ModelFinder function and selected according to the Bayesian Information Criterion (BIC). The chosen model was GTR + F + R10, which appropriately accounts for base frequency heterogeneity and rate variation across the different positions in the alignment. Tree visualization and annotation were conducted using the Interactive Tree Of Life (iTOL) v.7 [114].

### Gene expression analysis

Paired-end RNA-seq datasets were retrieved from the Sequence Read Archive using fastq-dump v2.8.1 [107]. Gene expression quantification, including raw counts and Transcripts Per Million (TPMs), were obtained using kallisto v0.44 [115] with default parameters. Customs R scripts (prep_expression_matrix.R and LMM_expression_analysis.R) available through GitHub (https://github.com/mariamarinr/LAR_Evolution/*)* were developed to prepare the expression data matrix and generate violin plots using the ggplot2 package v3.5.1 [116] illustrating the variation in gene expression (TPMs) across multiple samples [39]. These plots were specifically used to visualize the expression levels of *LAR* across specific plant tissues in different species, aiming to identify patterns in gene expression and to gain insights into potential functions. To assess the statistical significance of the difference in gene expression levels between the two *LAR* copies, a linear mixed-effects model (LMM) was employed. The model was formulated as:

$$TPM\_log\sim genes^\ast tissue+(1\vert sample)$$

where *TPM_log* represents the log-transformed transcript abundance; *genes* and *tissue* are fixed effects; and *sample* is included as a random effect to account for variability across biological replicates and sample-specific factors (e.g., physiological condition, developmental stage, or sequencing platform). This structure ensures more accurate estimates of fixed effects and reduces the risk of false positives. The model was fitted using restricted maximum likelihood (REML), and statistical significance for the fixed effects was assessed using t-tests with Satterthwaite’s method, as implemented in the lmerTest package v3.1–3 [117]. To further explore differences in gene expression across plant tissues and provide a more detailed evaluation of tissue-specific patterns for each *LAR* copy, a post-hoc pairwise comparison was conducted. This analysis was performed using the emmeans function v1.10.1 [118], which computed estimated marginal means (EMMs) [119] and pairwise contrasts for the interaction between genes and tissue. Specifically, pairwise comparisons were made for the genes within each level of the tissue factor, using t-tests to assess the significance of differences between the two *LAR* copies across the various tissues. To account for multiple comparisons, p-values were adjusted using the Bonferroni method. All statistical analyses were performed using R v4.3.3 [120].

### Functionalization analysis

In order to explore the functional divergence of the *LAR* gene copies in the same species, a screen for evidence of subfunctionalization and neofunctionalization was conducted. This involved comparing the sequence profiles of the two *LAR* copies to identify potential nonsynonymous substitutions that would lead to novel functions that had evolved since the duplication event. Specifically, we utilized sequence alignments using MAFFT v7.490 [121] and visualized in JalView v.2.11.4.1 [122] and ChimeraX v.1.8 [123] to detect differences in the amino acid positions within the LAR proteins, which could indicate changes in functional domains or active sites. Additionally, the alignments were subjected to an analysis with DivAAs (https://github.com/k-georgi/DivAAs) to identify specific amino acid positions that show systematic differences between the two groups of LAR sequences.

### Coexpression analysis

Coexpression analysis was performed to identify genes that present similarity in expression with the *LAR* genes of interest. A list with the *LAR* genes and a count table with normalized TPM values were used for this analysis. Pairwise Spearman correlation coefficients for gene expression values across all samples were calculated for each *LAR* gene in the list. The coexpressed genes were assessed based on their Spearman correlation values, adjusted p-values, and total expression level. Genes with low expression levels were excluded, and only gene pairs with a correlation coefficient > 0.65 and an adjusted p-value < 0.05 were considered significant. This coexpression analysis was implemented in a Python script - coexp3.py [51]. The functional annotation of the genes belonging to *V. vinifera* (Vvinifera_457_v2.1), *T. cacao* (Tcacao_523_v2.1), and *M. oleifera* (GCA_021397835.1) were derived from *M. truncatula* and *A. thaliana* using the Python script - construct_anno.py [51] available at: https://github.com/bpucker/ApiaceaeFNS1.

### Identification of cis-regulatory elements (CREs) in **LAR** promoters

To investigate the differences in the regulatory mechanisms of *LAR1* and *LAR2* gene copies, the promoter region of each gene was extracted and analyzed for cis-regulatory elements. Promoter and transposable element (TEs) analyses were performed for *V. vinifera*, *T. cacao*, and *M. oleifera*, which were selected to represent distinct eudicot lineages (*Vitales*,* Malvales*,* Brassicales*, respectively) and to ensure the use of high-quality genome assemblies with reliable gene annotations. For *V. vinifera*, the *LAR1* and *LAR2* promoters previously reported by [86] were used, available in GenBank under accession numbers MT586116 and MT586117, respectively. For *T. cacao* and *M. oleifera*, paired-end RNA-seq reads were retrieved from the Sequence Read Archive (SRA) using fasterq-dump v.3.0.3 [124]. The complete list of SRR accessions is provided in [39]. To ensure accuracy in the identification of the transcription start sites (TSS), an alignment of these reads to their respective genome assemblies was performed using HISAT v2.2.1 [109], a splice-aware aligner optimized for short RNA-seq reads. This mapping workflow was automated using a custom Bash script (hisat2_mapping.sh) available at the GitHub repository: https://github.com/mariamarinr/LAR_Evolution/. This pipeline also handled the conversion, merging, sorting, and indexing of alignment files with Samtools v1.15 [110] for each species. The resulting sorted BAM files (available at [ 39 ]) were visualized using the Integrative Genomics Viewer (IGV) [125] to identify the regions with transcriptional activity upstream of LAR encoding sequences. Based on RNA-seq coverage patterns, the transcription start site (TSS) was inferred for each *LAR* gene copy, and promoter regions were defined as approximately 1500 bp upstream of the TSS. These promoter sequences were extracted from the genome assemblies using BEDtools v2.30 [126] (See additional file 4 [ 39 ]) and analyzed for potential cis-regulatory elements using the PLACE database [40] at https://www.dna.affrc.go.jp/PLACE/.

### Identification of transposable elements (TEs)

To evaluate whether transposable elements (TEs) may contribute to the regulation of *LAR* gene expression, genome-wide annotation of TEs was performed for the species: *V. vinifera*, *T. cacao*, and *M. oleifera*. TE annotation was conducted for each genome sequence using the Extensive *de-novo* TE Annotator (EDTA) v.2.2.2 [127]. EDTA was run in sensitive mode with both annotation and evaluation options enabled to ensure a complete detection of both abundant and low-copy TEs. To identify overlaps between TEs and *LAR* loci, the intersect function of BEDTools v2.30 [126] was employed. This approach was intended to reveal TE insertions located within or adjacent to *LAR* genes or their promoter regions across the three species. Each overlapping TE was classified by type (e.g., LTR retrotransposons, TIR DNA transposons), and family, allowing further exploration of whether particular TE lineages were commonly linked with **LAR** regulatory regions. The complete list of overlapping TE insertions and their annotations is provided in [39].

### Synteny analysis

Synteny analysis using JCVI/MCscan [128] was performed to visualize conserved genomic regions across the genome sequences of multiple species (Table 1). Flanking regions surrounding the identified *LAR* loci, typically including 10 to 15 protein-coding genes upstream and downstream, were manually selected based on their physical proximity to *LAR* and the preservation of local gene order, serving as reference points. Gene connections between species were manually validated and revised by comparing the predicted syntenic blocks with the phylogenetic relationships reported by [105], ensuring consistency between gene conservation and species divergence. To identify homologous genes or syntenic regions that might be missing in current annotations, BLAST v.2.13.0 [129] databases were constructed for multiple species using the ‘makeblastdb’ command, specifying nucleotide sequence types. Query sequences were then searched against these databases using ‘tblastn’.

**Table 1:** List of species used for synteny analysis

Species	Family	Order	Data set ID	Database	Reference
*Vitis vinifera*	*Vitaceae*	*Vitales*	Vvinifera_457_v2.1	Phytozome	[130]
*Theobroma cacao*	*Malvaceae*	*Malvales*	Tcacao_523_v2.1	Phytozome	[131]
*Bretschneidera sinensis*	*Akaniaceae*	*Brassicales*	GCA_018105755.1	NCBI	[132]
*Moringa oleifera*	*Moringaceae*	*Brassicales*	GCA_021397835.1	NCBI	[133]
*Carica papaya*	*Caricaceae*	*Brassicales*	Cpapaya_113_ASGPBv0.4	Phytozome	[134]
*Eutrema salsugineum*	*Brassicaceae*	*Brassicales*	Esalsugineum_173_v1.0	Phytozome	[135]
*Capsella rubella*	*Brassicaceae*	*Brassicales*	Crubella_474_v1.1	Phytozome	[136]
*Boechera stricta*	*Brassicaceae*	*Brassicales*	Bstricta_278_ v1.2	Phytozome	[137]
*Arabidopsis halleri*	*Brassicaceae*	*Brassicales*	Ahalleri_765_v2.1.0	Phytozome	[138]
*Arabidopsis thaliana*	*Brassicaceae*	*Brassicales*	Athaliana_447_Araport11	Phytozome	[139, 140]
*Brassica rapa*	*Brassicaceae*	*Brassicales*	GCA_003434825.1	NCBI	[141]
*Cleome gynandra*	*Cleomaceae*	*Brassicales*	GCA_036759665.1	NCBI	[142]
*Aethionema arabicum*	*Brassicaceae*	*Brassicales*	Ae.arabicum_v3.1	-	[143]

## Data Availability

All the genomic, transcriptomic and annotation data used in this study was obtained from the NCBI Sequence Read Archive (SRA) and Phytozome. The data charts supporting the results and conclusions are included in the article and additional files. Scripts used in the different analyses have been deposited in our GitHub repository (https://github.com/mariamarinr/LAR_Evolution/) and all sequences and additional files can be found online via bonndata (10.60507/FK2/UKAAO0).

## References

[1] Singh S, Kariyat RR. Exposure to polyphenol-rich purple corn pericarp extract restricts fall armyworm (Spodoptera frugiperda) growth. Plant Signal Behav. 2020;15:1784545. 10.1080/15592324.2020.1784545. 32580616 10.1080/15592324.2020.1784545 PMC8550206

[2] War AR, Paulraj MG, Ahmad T, Buhroo AA, Hussain B, Ignacimuthu S, et al. Mechanisms of plant defense against insect herbivores. Plant Signal Behav. 2012;7:1306–20. 10.4161/psb.21663. 22895106 10.4161/psb.21663 PMC3493419

[3] Yu K, Song Y, Lin J, Dixon RA. The complexities of Proanthocyanidin biosynthesis and its regulation in plants. Plant Commun. 2022;4:100498. 10.1016/j.xplc.2022.100498. 36435967 10.1016/j.xplc.2022.100498 PMC10030370

[4] Maisuria VB, Okshevsky M, Déziel E, Tufenkji N. Proanthocyanidin interferes with intrinsic antibiotic resistance mechanisms of Gram-Negative bacteria. Adv Sci. 2019;6:1802333. 10.1002/advs.201802333. 10.1002/advs.201802333 PMC6685479 31406662

[5] Maisuria VB, Los Santos YL, Tufenkji N, Déziel E. Cranberry-derived proanthocyanidins impair virulence and inhibit quorum sensing of Pseudomonas aeruginosa. Sci Rep. 2016;6:30169. 10.1038/srep30169. 27503003 10.1038/srep30169 PMC4977528

[6] Ulrey RK, Barksdale SM, Zhou W, van Hoek ML. Cranberry proanthocyanidins have anti-biofilm properties against Pseudomonas aeruginosa. BMC Complement Altern Med. 2014;14:499. 10.1186/1472-6882-14-499. 25511463 10.1186/1472-6882-14-499 PMC4320558

[7] Ullah C, Unsicker SB, Reichelt M, Gershenzon J, Hammerbacher A. Accumulation of Catechin and proanthocyanidins in black Poplar stems after infection by plectosphaerella populi: hormonal Regulation, biosynthesis and antifungal activity. Front Plant Sci. 2019;10. 10.3389/fpls.2019.01441. 10.3389/fpls.2019.01441 PMC6873352 31803202

[8] Lazarus SA, Adamson GE, Hammerstone JF, Schmitz HH. High-Performance liquid Chromatography/Mass spectrometry analysis of proanthocyanidins in foods and beverages. J Agric Food Chem. 1999;47:3693–701. 10.1021/jf9813642. 10552707 10.1021/jf9813642

[9] Santos-Buelga C, Scalbert A. Proanthocyanidins and tannin-like compounds – nature, occurrence, dietary intake and effects on nutrition and health. J Sci Food Agric. 2000;80:1094–117. 10.1002/(SICI)1097-0010(20000515)80:7%3C1094::AID-JSFA569%3E3.0.CO;2-1.

[10] Xie Q, Xu Z. Sustainable agriculture: from sweet sorghum planting and ensiling to ruminant feeding. Mol Plant. 2019;12:603–6. 10.1016/j.molp.2019.04.001. 31002980 10.1016/j.molp.2019.04.001

[11] Todd JJ, Vodkin LO. Pigmented soybean (Glycine max) seed coats accumulate proanthocyanidins during development. Plant Physiol. 1993;102:663–70. 10.1104/pp.102.2.663. 12231856 10.1104/pp.102.2.663 PMC158826

[12] Debeaujon I, Nesi N, Perez P, Devic M, Grandjean O, Caboche M, et al. Proanthocyanidin-Accumulating cells in Arabidopsis testa: regulation of differentiation and role in seed development. Plant Cell. 2003;15:2514–31. 10.1105/tpc.014043. 14555692 10.1105/tpc.014043 PMC280558

[13] Clegg MT, Durbin ML. Flower color variation: A model for the experimental study of evolution. Proc Natl Acad Sci. 2000;97:7016–23. 10.1073/pnas.97.13.7016. 10860965 10.1073/pnas.97.13.7016 PMC34378

[14] Debeaujon I, Lepiniec L, Pourcel L, Routaboul J-M. Seed coat development and dormancy. Annual plant reviews volume 27: seed Development, dormancy and germination. John Wiley & Sons, Ltd; 2007. pp. 25–49. 10.1002/9780470988848.ch2.

[15] Barry TN, Mcnabb WC, Kemp PD, Waghorn GC, Min BR, Luque A. The effect of condensed tannins in Lotus corniculatus upon reproductive efficiency and wool production in sheep during late summer and autumn. 1999. 10.33584/jnzg.1999.61.2354

[16] Aerts RJ, Barry TN, McNabb WC. Polyphenols and agriculture: beneficial effects of proanthocyanidins in forages. Agric Ecosyst Environ. 1999;75:1–12. 10.1016/S0167-8809(99)00062-6.

[17] Koes RE, Quattrocchio F, Mol JNM. The flavonoid biosynthetic pathway in plants: function and evolution. BioEssays. 1994;16:123–32. 10.1002/bies.950160209.

[18] Winkel-Shirley B. It takes a Garden. How work on diverse plant species has contributed to an Understanding of flavonoid metabolism. Plant Physiol. 2001;127:1399–404. 11743081 PMC1540170

[19] Winkel-Shirley B, Flavonoid Biosynthesis. A colorful model for Genetics, Biochemistry, cell Biology, and biotechnology. Plant Physiol. 2001;126:485–93. 10.1104/pp.126.2.485. 11402179 10.1104/pp.126.2.485 PMC1540115

[20] Liu C, Wang X, Shulaev V, Dixon RA. A role for Leucoanthocyanidin reductase in the extension of proanthocyanidins. Nat Plants. 2016;2:16182. 10.1038/nplants.2016.182. 27869786 10.1038/nplants.2016.182

[21] Jun JH, Xiao X, Rao X, Dixon RA. Proanthocyanidin subunit composition determined by functionally diverged dioxygenases. Nat Plants. 2018;4:1034–43. 10.1038/s41477-018-0292-9. 30478357 10.1038/s41477-018-0292-9

[22] Bogs J, Downey MO, Harvey JS, Ashton AR, Tanner GJ, Robinson SP. Proanthocyanidin synthesis and expression of genes encoding Leucoanthocyanidin reductase and Anthocyanidin reductase in developing grape berries and grapevine leaves. Plant Physiol. 2005;139:652–63. 10.1104/pp.105.064238. 16169968 10.1104/pp.105.064238 PMC1255985

[23] Liu Y, Shi Z, Maximova S, Payne MJ, Guiltinan MJ. Proanthocyanidin synthesis in theobroma cacao: genes encoding Anthocyanidin synthase, Anthocyanidin reductase, and Leucoanthocyanidin reductase. BMC Plant Biol. 2013;13:202. 10.1186/1471-2229-13-202. 24308601 10.1186/1471-2229-13-202 PMC4233638

[24] Wang X, Yuan B, Zhu N, Mu R, Zheng H, Shao C, et al. Identification and functional analysis of the promoter of a Leucoanthocyanidin reductase gene from Gossypium hirsutum. Mol Biotechnol. 2023;65:645–54. 10.1007/s12033-022-00571-4. 36155889 10.1007/s12033-022-00571-4

[25] Henry-Kirk RA, McGhie TK, Andre CM, Hellens RP, Allan AC. Transcriptional analysis of Apple fruit Proanthocyanidin biosynthesis. J Exp Bot. 2012;63:5437. 10.1093/jxb/ers193. 22859681 10.1093/jxb/ers193 PMC3444262

[26] Yang Y, Yao G, Yue W, Zhang S, Wu J. Transcriptome profiling reveals differential gene expression in Proanthocyanidin biosynthesis associated with red/green skin color mutant of Pear (Pyrus communis L). Front Plant Sci. 2015;6. 10.3389/fpls.2015.00795. 10.3389/fpls.2015.00795 PMC4588701 26483812

[27] Wang P, Zhang L, Jiang X, Dai X, Xu L, Li T, et al. Evolutionary and functional characterization of Leucoanthocyanidin reductases from camellia sinensis. Planta. 2018;247:139–54. 10.1007/s00425-017-2771-z. 28887677 10.1007/s00425-017-2771-z PMC5756577

[28] Pucker B, Reiher F, Schilbert HM. Automatic identification of players in the flavonoid biosynthesis with application on the biomedicinal plant *Croton tiglium*. Plants. 2020;9:1103. 10.3390/plants9091103. 32867203 10.3390/plants9091103 PMC7570183

[29] Shirley BW, Kubasek WL, Storz G, Bruggemann E, Koornneef M, Ausubel FM, et al. Analysis of Arabidopsis mutants deficient in flavonoid biosynthesis. Plant J. 1995;8:659–71. 10.1046/j.1365-313X.1995.08050659.x. 8528278 10.1046/j.1365-313x.1995.08050659.x

[30] Abrahams S, Lee E, Walker AR, Tanner GJ, Larkin PJ, Ashton AR. The Arabidopsis TDS4 gene encodes Leucoanthocyanidin dioxygenase (LDOX) and is essential for Proanthocyanidin synthesis and vacuole development. Plant J. 2003;35:624–36. 10.1046/j.1365-313X.2003.01834.x. 12940955 10.1046/j.1365-313x.2003.01834.x

[31] Zhao J, Pang Y, Dixon RA. The mysteries of Proanthocyanidin transport and polymerization. Plant Physiol. 2010;153:437–43. 10.1104/pp.110.155432. 20388668 10.1104/pp.110.155432 PMC2879784

[32] Lepiniec L, Debeaujon I, Routaboul J-M, Baudry A, Pourcel L, Nesi N, et al. Genetics and biochemistry of seed flavonoids. Annu Rev Plant Biol. 2006;57:405–30. 10.1146/annurev.arplant.57.032905.105252. 16669768 10.1146/annurev.arplant.57.032905.105252

[33] Ramsay NA, Glover BJ. MYB–bHLH–WD40 protein complex and the evolution of cellular diversity. Trends Plant Sci. 2005;10:63–70. 10.1016/j.tplants.2004.12.011. 15708343 10.1016/j.tplants.2004.12.011

[34] Devic M, Guilleminot J, Debeaujon I, Bechtold N, Bensaude E, Koornneef M, et al. The BANYULS gene encodes a DFR-like protein and is a marker of early seed coat development. Plant J. 1999;19:387–98. 10.1046/j.1365-313X.1999.00529.x. 10504561 10.1046/j.1365-313x.1999.00529.x

[35] Xie D-Y, Sharma SB, Paiva NL, Ferreira D, Dixon RA. Role of Anthocyanidin Reductase, encoded by BANYULS in plant flavonoid biosynthesis. Science. 2003;299:396–9. 10.1126/science.1078540. 12532018 10.1126/science.1078540

[36] Ranwez V, Douzery EJP, Cambon C, Chantret N, Delsuc F. MACSE v2: toolkit for the alignment of coding sequences accounting for frameshifts and stop codons. Mol Biol Evol. 2018;35:2582–4. 10.1093/molbev/msy159. 30165589 10.1093/molbev/msy159 PMC6188553

[37] Nguyen L-T, Schmidt HA, von Haeseler A, Minh BQ. IQ-TREE: A fast and effective stochastic algorithm for estimating Maximum-Likelihood phylogenies. Mol Biol Evol. 2015;32:268–74. 10.1093/molbev/msu300. 25371430 10.1093/molbev/msu300 PMC4271533

[38] Minh BQ, Schmidt HA, Chernomor O, Schrempf D, Woodhams MD, von Haeseler A, et al. IQ-TREE 2: new models and efficient methods for phylogenetic inference in the genomic era. Mol Biol Evol. 2020;37:1530–4. 10.1093/molbev/msaa015. 32011700 10.1093/molbev/msaa015 PMC7182206

[39] Marín Recinos MF, Pucker B. Supplementary data to evolutionary dynamics of the Proanthocyanidin biosynthesis gene LAR. 2025. 10.60507/FK2/UKAAO0 10.1186/s12864-025-12429-5 PMC12831355 41420151

[40] Higo K, Ugawa Y, Iwamoto M, Korenaga T. Plant cis-acting regulatory DNA elements (PLACE) database: 1999. Nucleic Acids Res. 1999;27:297–300. 10.1093/nar/27.1.297. 9847208 10.1093/nar/27.1.297 PMC148163

[41] Quattrocchio F, Wing JF, Leppen HTC, Mol JNM, Koes RE. Regulatory genes controlling anthocyanin pigmentation are functionally conserved among plant species and have distinct sets of Target Genes.:17. 10.1105/tpc.5.11.1497 10.1105/tpc.5.11.1497 PMC160381 12271045

[42] Cheng A-X, Zhang X, Han X-J, Zhang Y-Y, Gao S, Liu C-J, et al. Identification of chalcone isomerase in the basal land plants reveals an ancient evolution of enzymatic cyclization activity for synthesis of flavonoids. New Phytol. 2018;217:909–24. 10.1111/nph.14852. 29083033 10.1111/nph.14852

[43] Choudhary N, Pucker B. Conserved amino acid residues and gene expression patterns associated with the substrate preferences of the competing enzymes FLS and DFR. PLoS ONE. 2024;19:e0305837. 10.1371/journal.pone.0305837. 39196921 10.1371/journal.pone.0305837 PMC11356453

[44] Yang L, Zhang S, Chu D, Wang X. Exploring the evolution of CHS gene family in plants. Front Genet. 2024;15. 10.3389/fgene.2024.1368358. 10.3389/fgene.2024.1368358 PMC11091334 38746055

[45] Yonekura-Sakakibara K, Higashi Y, Nakabayashi R. The origin and evolution of plant flavonoid metabolism. Front Plant Sci. 2019;10:943. 10.3389/fpls.2019.00943. 31428108 10.3389/fpls.2019.00943 PMC6688129

[46] Davies KM, Jibran R, Zhou Y, Albert NW, Brummell DA, Jordan BR, et al. The evolution of flavonoid biosynthesis: A bryophyte perspective. Front Plant Sci. 2020;11. 10.3389/fpls.2020.00007. 10.3389/fpls.2020.00007 PMC7010833 32117358

[47] Marin-Recinos MF, Pucker B. Genetic factors explaining anthocyanin pigmentation differences. BMC Plant Biol. 2024;24:627. 10.1186/s12870-024-05316-w. 38961369 10.1186/s12870-024-05316-w PMC11221117

[48] Rausher, M. D. 2006. The evolution of flavonoids and their genes. in Grotewold, E. (ed.), The Science of Flavonoids. Springer. 10.1007/978-0-387-28822-2_7

[49] Stafford HA. Flavonoid evolution: an enzymic approach. Plant Physiol. 1991;96:680–5. 10.1104/pp.96.3.680. 16668242 10.1104/pp.96.3.680 PMC1080830

[50] Wheeler LC, Dunbar-Wallis A, Schutz K, Smith SD. Evolutionary walks through flower colour space driven by gene expression in Petunia and allies (Petunieae). Proc R Soc B Biol Sci. 2023;290:20230275. 10.1098/rspb.2023.0275. 10.1098/rspb.2023.0275 PMC10320354 37403504

[51] Pucker B, Iorizzo M. Apiaceae FNS i originated from F3H through tandem gene duplication. PLoS One. 2023;18:e0280155. 10.1371/journal.pone.0280155. 36656808 10.1371/journal.pone.0280155 PMC9851555

[52] Khoo HE, Azlan A, Tang ST, Lim SM. Anthocyanidins and anthocyanins: colored pigments as food, pharmaceutical ingredients, and the potential health benefits. Food Nutr Res. 2017. 10.1080/16546628.2017.1361779 10.1080/16546628.2017.1361779 PMC5613902 28970777

[53] Lois R. Accumulation of UV-absorbing flavonoids induced by UV-B radiation in *Arabidopsis Thaliana* L.: I. Mechanisms of UV-resistance in Arabidopsis. Planta. 1994;194:498–503.

[54] Miller R, Owens SJ, Rørslett B. Plants and colour: flowers and pollination. Opt Laser Technol. 2011;43:282–94. 10.1016/j.optlastec.2008.12.018.

[55] Wheeler LC, Walker JF, Ng J, Deanna R, Dunbar-Wallis A, Backes A, et al. Transcription factors evolve faster than their structural gene targets in the flavonoid pigment pathway. Mol Biol Evol. 2022;39:msac044. 10.1093/molbev/msac044. 35212724 10.1093/molbev/msac044 PMC8911815

[56] Güngör E, Brouwer P, Dijkhuizen LW, Shaffar DC, Nierop KGJ, de Vos RCH, et al. Azolla ferns testify: seed plants and ferns share a common ancestor for Leucoanthocyanidin reductase enzymes. New Phytol. 2021;229:1118–32. 10.1111/nph.16896. 32858769 10.1111/nph.16896 PMC7820995

[57] Basu R, Dutta S, Pal A, Sengupta M, Chattopadhyay S. Calmodulin7: recent insights into emerging roles in plant development and stress. Plant Mol Biol. 2021;107:1–20. 10.1007/s11103-021-01177-1. 34398355 10.1007/s11103-021-01177-1

[58] Chutipaijit S, Cha-um S, Sompornpailin K. High contents of proline and anthocyanin increase protective response to salinity in *Oryza sativa* L. spp. *Indica*. Aust J Crop Sci. 2011;5:1191–8.

[59] Ishikawa T, Watanabe N, Nagano M, Kawai-Yamada M, Lam E. Bax inhibitor-1: a highly conserved Endoplasmic reticulum-resident cell death suppressor. Cell Death Differ. 2011;18:1271–8. 10.1038/cdd.2011.59. 21597463 10.1038/cdd.2011.59 PMC3172100

[60] Nakajima K, Furutani I, Tachimoto H, Matsubara H, Hashimoto T. SPIRAL1 encodes a Plant-Specific Microtubule-Localized protein required for directional control of rapidly expanding Arabidopsis Cells[W]. Plant Cell. 2004;16:1178–90. 10.1105/tpc.017830. 15084720 10.1105/tpc.017830 PMC423208

[61] Das M, Haberer G, Panda A, Das Laha S, Ghosh TC, Schäffner AR. Expression pattern similarities support the prediction of orthologs retaining common functions after gene duplication events. Plant Physiol. 2016;171:2343–57. 10.1104/pp.15.01207. 27303025 10.1104/pp.15.01207 PMC4972257

[62] Noble JA, Bielski NV, Liu M-CJ, DeFalco TA, Stegmann M, Nelson ADL, et al. Evolutionary analysis of the LORELEI gene family in plants reveals regulatory subfunctionalization. Plant Physiol. 2022;190:2539–56. 10.1093/plphys/kiac444. 36156105 10.1093/plphys/kiac444 PMC9706458

[63] Maugé C, Granier T, d’Estaintot BL, Gargouri M, Manigand C, Schmitter J-M, et al. Crystal structure and catalytic mechanism of Leucoanthocyanidin reductase from *Vitis vinifera*. J Mol Biol. 2010;397:1079–91. 10.1016/j.jmb.2010.02.002. 20138891 10.1016/j.jmb.2010.02.002

[64] Bartlett GJ, Porter CT, Borkakoti N, Thornton JM. Analysis of catalytic residues in enzyme active sites. J Mol Biol. 2002;324:105–21. 10.1016/S0022-2836(02)01036-7. 12421562 10.1016/s0022-2836(02)01036-7

[65] Mattos C, Ringe D. Locating and characterizing binding sites on proteins. Nat Biotechnol. 1996;14:595–9. 10.1038/nbt0596-595. 9630949 10.1038/nbt0596-595

[66] Brzovic PS, Kayastha AM, Miles EW, Dunn MF. Substitution of glutamic acid 109 by aspartic acid alters the substrate specificity and catalytic activity of the.beta.-subunit in the Tryptophan synthase bienzyme complex from Salmonella typhimurium. Biochemistry. 1992;31:1180–90. 10.1021/bi00119a030. 1346502 10.1021/bi00119a030

[67] Peterson CB, Burman DL, Schachman HK. Effects of replacement of active site residue glutamine 231 on activity and allosteric properties of aspartate transcarbamoylase. Biochemistry. 1992;31:8508–15. 10.1021/bi00151a018. 1390636 10.1021/bi00151a018

[68] Roesgaard MA, Lundsgaard JE, Newcombe EA, Jacobsen NL, Pesce F, Tranchant EE, et al. Deciphering the alphabet of Disorder—Glu and asp act differently on local but not global properties. Biomolecules. 2022;12:1426. 10.3390/biom12101426. 36291634 10.3390/biom12101426 PMC9599281

[69] Wilson RH, Zamfir S, Sumner I. Molecular dynamics simulations reveal a new role for a conserved active site asparagine in a ubiquitin-conjugating enzyme. J Mol Graph Model. 2017;76:403–11. 10.1016/j.jmgm.2017.07.006. 28772203 10.1016/j.jmgm.2017.07.006

[70] Zhou H-X, Pang X. Electrostatic interactions in protein Structure, Folding, Binding, and condensation. Chem Rev. 2018;118:1691–741. 10.1021/acs.chemrev.7b00305. 29319301 10.1021/acs.chemrev.7b00305 PMC5831536

[71] Taylor JC, Takusagawa F, Markham GD. The active site loop of S-Adenosylmethionine synthetase modulates catalytic efficiency. Biochemistry. 2002;41:9358–69. 10.1021/bi025851t. 12135357 10.1021/bi025851t

[72] Zhou H-X, Wong K-Y, Vijayakumar M. Design of fast enzymes by optimizing interaction potential in active site. Proc Natl Acad Sci. 1997;94:12372–7. 10.1073/pnas.94.23.12372. 9356456 10.1073/pnas.94.23.12372 PMC24950

[73] Davies KM, Andre CM, Kulshrestha S, Zhou Y, Schwinn KE, Albert NW, et al. The evolution of flavonoid biosynthesis. Philos Trans R Soc B Biol Sci. 2024;379:20230361. 10.1098/rstb.2023.0361. 10.1098/rstb.2023.0361 PMC11528363 39343026

[74] Dixon RA, Xie D, Sharma SB. Proanthocyanidins – a final frontier in flavonoid research? New Phytol. 2005;165:9–28. 10.1111/j.1469-8137.2004.01217.x. 15720617 10.1111/j.1469-8137.2004.01217.x

[75] He F, Pan Q-H, Shi Y, Duan C-Q. Biosynthesis and genetic regulation of proanthocyanidins in plants. Molecules. 2008;13:2674. 10.3390/molecules13102674. 18971863 10.3390/molecules13102674 PMC6245171

[76] Quattrocchio F, Baudry A, Lepiniec L, Grotewold E. The regulation of flavonoid biosynthesis. New York, NY: Springer; 2006. 10.1007/978-0-387-28822-2_4. :pp97-122.

[77] Dubos C, Stracke R, Grotewold E, Weisshaar B, Martin C, Lepiniec L. MYB transcription factors in Arabidopsis. Trends Plant Sci. 2010;15:573–81. 10.1016/j.tplants.2010.06.005. 20674465 10.1016/j.tplants.2010.06.005

[78] Stracke R, Werber M, Weisshaar B. The R2R3-MYB gene family in *Arabidopsis Thaliana*. Curr Opin Plant Biol. 2001;4:447–56. 10.1016/S1369-5266(00)00199-0. 11597504 10.1016/s1369-5266(00)00199-0

[79] Bogs J, Jaffé FW, Takos AM, Walker AR, Robinson SP. The grapevine transcription factor VvMYBPA1 regulates Proanthocyanidin synthesis during fruit development. Plant Physiol. 2007;143:1347–61. 10.1104/pp.106.093203. 17208963 10.1104/pp.106.093203 PMC1820911

[80] Liu C, Jun JH, Dixon RA. MYB5 and MYB14 play pivotal roles in seed coat polymer biosynthesis in medicago truncatula. Plant Physiol. 2014;165:1424–39. 10.1104/pp.114.241877. 24948832 10.1104/pp.114.241877 PMC4119029

[81] Terrier N, Torregrosa L, Ageorges A, Vialet S, Verriès C, Cheynier V, et al. Ectopic expression of VvMybPA2 promotes Proanthocyanidin biosynthesis in grapevine and suggests additional targets in the pathway. Plant Physiol. 2009;149:1028–41. 10.1104/pp.108.131862. 19098092 10.1104/pp.108.131862 PMC2633825

[82] Qian Y, Zhang T, Yu Y, Gou L, Yang J, Xu J, et al. Regulatory mechanisms of bHLH transcription factors in plant adaptive responses to various abiotic stresses. Front Plant Sci. 2021;12:677611. 10.3389/fpls.2021.677611. 34220896 10.3389/fpls.2021.677611 PMC8250158

[83] Hichri I, Barrieu F, Bogs J, Kappel C, Delrot S, Lauvergeat V. Recent advances in the transcriptional regulation of the flavonoid biosynthetic pathway. J Exp Bot. 2011;62:2465–83. 10.1093/jxb/erq442. 21278228 10.1093/jxb/erq442

[84] Hichri I, Heppel SC, Pillet J, Léon C, Czemmel S, Delrot S, et al. The basic Helix-Loop-Helix transcription factor MYC1 is involved in the regulation of the flavonoid biosynthesis pathway in grapevine. Mol Plant. 2010;3:509–23. 10.1093/mp/ssp118. 20118183 10.1093/mp/ssp118

[85] Matus JT, Poupin MJ, Cañón P, Bordeu E, Alcalde JA, Arce-Johnson P. Isolation of WDR and bHLH genes related to flavonoid synthesis in grapevine (Vitis vinifera L). Plant Mol Biol. 2010;72:607–20. 10.1007/s11103-010-9597-4. 20112051 10.1007/s11103-010-9597-4

[86] Cheng J, Yu K, Zhang M, Shi Y, Duan C, Wang J. The effect of light intensity on the expression of Leucoanthocyanidin reductase in grapevine calluses and analysis of its promoter activity. Genes. 2020;11:1156. 10.3390/genes11101156. 33007888 10.3390/genes11101156 PMC7600843

[87] Dixon RA, Sarnala S. Proanthocyanidin Biosynthesis—a matter of protection. Plant Physiol. 2020;184:579–91. 10.1104/pp.20.00973. 32817234 10.1104/pp.20.00973 PMC7536678

[88] Solano R, Nieto C, Avila J, Cañas L, Diaz I, Paz-Ares J. Dual DNA binding specificity of a petal epidermis-specific MYB transcription factor (MYB.Ph3) from Petunia hybrida. EMBO J. 1995;14:1773–84. 10.1002/j.1460-2075.1995.tb07166.x. 7737128 10.1002/j.1460-2075.1995.tb07166.x PMC398271

[89] Gubler F, Kalla R, Roberts JK, Jacobsen JV. Gibberellin-regulated expression of a Myb gene in barley aleurone cells: evidence for Myb transactivation of a high-pI alpha-amylase gene promoter. Plant Cell. 1995;7:1879–91. 10.1105/tpc.7.11.1879. 8535141 10.1105/tpc.7.11.1879 PMC161046

[90] Mutasa-Göttgens E, Hedden P. Gibberellin as a factor in floral regulatory networks. J Exp Bot. 2009;60:1979–89. 10.1093/jxb/erp040. 19264752 10.1093/jxb/erp040

[91] Singh DP, Jermakow AM, Swain SM. Gibberellins are required for seed development and pollen tube growth in Arabidopsis. Plant Cell. 2002;14:3133–47. 10.1105/tpc.003046. 12468732 10.1105/tpc.003046 PMC151207

[92] Bate N, Twell D. Functional architecture of a late pollen promoter: pollen-specific transcription is developmentally regulated by multiple stage-specific and co-dependent activator elements. Plant Mol Biol. 1998;37:859–69. 9678581 10.1023/a:1006095023050

[93] Moyano E, Martínez-Garcia JF, Martin C. Apparent redundancy in Myb gene function provides gearing for the control of flavonoid biosynthesis in antirrhinum flowers. Plant Cell. 1996;8:1519–32. 10.1105/tpc.8.9.1519. 8837506 10.1105/tpc.8.9.1519 PMC161295

[94] Lisch D. How important are transposons for plant evolution? Nat Rev Genet. 2013;14:49–61. 10.1038/nrg3374. 23247435 10.1038/nrg3374

[95] Butelli E, Licciardello C, Zhang Y, Liu J, Mackay S, Bailey P, et al. Retrotransposons control Fruit-Specific, Cold-Dependent accumulation of anthocyanins in blood oranges. Plant Cell. 2012;24:1242–55. 10.1105/tpc.111.095232. 22427337 10.1105/tpc.111.095232 PMC3336134

[96] This P, Lacombe T, Cadle-Davidson M, Owens CL. Wine grape (Vitis vinifera L.) color associates with allelic variation in the domestication gene VvmybA1. Theor Appl Genet. 2007;114:723–30. 10.1007/s00122-006-0472-2. 17221259 10.1007/s00122-006-0472-2

[97] Tian Y, Thrimawithana A, Ding T, Guo J, Gleave A, Chagné D, et al. Transposon insertions regulate genome-wide allele-specific expression and underpin flower colour variations in Apple (Malus spp). Plant Biotechnol J. 2022;20:1285–97. 10.1111/pbi.13806. 35258172 10.1111/pbi.13806 PMC9241373

[98] Liu X, Lu Y, Yan M, Sun D, Hu X, Liu S, et al. Genome-Wide Identification, Localization, and expression analysis of Proanthocyanidin-Associated genes in brassica. Front Plant Sci. 2016;7. 10.3389/fpls.2016.01831. 10.3389/fpls.2016.01831 PMC5145881 28018375

[99] Lu N, Jun JH, Li Y, Dixon RA. An unconventional Proanthocyanidin pathway in maize. Nat Commun. 2023;14:4349. 10.1038/s41467-023-40014-5. 37468488 10.1038/s41467-023-40014-5 PMC10356931

[100] Brockington SF, Yang Y, Gandia-Herrero F, Covshoff S, Hibberd JM, Sage RF, et al. Lineage-specific gene radiations underlie the evolution of novel betalain pigmentation in caryophyllales. New Phytol. 2015;207:1170–80. 10.1111/nph.13441. 25966996 10.1111/nph.13441 PMC4557044

[101] Pucker B, Walker-Hale N, Dzurlic J, Yim WC, Cushman JC, Crum A, et al. Multiple mechanisms explain loss of anthocyanins from betalain-pigmented Caryophyllales, including repeated wholesale loss of a key Anthocyanidin synthesis enzyme. New Phytol. 2023. 10.1111/nph.19341. ;n/a n/a. 37897060 10.1111/nph.19341 PMC10952170

[102] Wu X, Huang H, Childs H, Wu Y, Yu L, Pehrsson P PR. Glucosinolates in brassica vegetables: characterization and factors that influence Distribution, Content, and intake. Annu Rev Food Sci Technol. 2021;12(12, 2021):485–511. 10.1146/annurev-food-070620-025744. 33467908 10.1146/annurev-food-070620-025744

[103] Lu N. Revisiting decade-old questions in Proanthocyanidin biosynthesis: current Understanding and new challenges. Front Plant Sci. 2024;15. 10.3389/fpls.2024.1373975. 10.3389/fpls.2024.1373975 PMC11002137 38595764

[104] Rempel A, Choudhary N, Pucker B. KIPEs3: automatic annotation of biosynthesis pathways. PLoS ONE. 2023;18:e0294342. 10.1371/journal.pone.0294342. 37972102 10.1371/journal.pone.0294342 PMC10653506

[105] Li H-T, Luo Y, Gan L, Ma P-F, Gao L-M, Yang J-B, et al. Plastid phylogenomic insights into relationships of all flowering plant families. BMC Biol. 2021;19:232. 10.1186/s12915-021-01166-2. 34711223 10.1186/s12915-021-01166-2 PMC8555322

[106] Gabriel L, Brůna T, Hoff KJ, Ebel M, Lomsadze A, Borodovsky M et al. BRAKER3: Fully Automated Genome Annotation Using RNA-Seq and Protein Evidence with GeneMark-ETP, AUGUSTUS and TSEBRA. 2023;:2023.06.10.544449. 10.1101/2023.06.10.544449 10.1101/gr.278090.123 PMC11216308 38866550

[107] Katz K, Shutov O, Lapoint R, Kimelman M, Brister JR, O’Sullivan C. The sequence read archive: a decade more of explosive growth. Nucleic Acids Res. 2022;50:D387–90. 10.1093/nar/gkab1053. 34850094 10.1093/nar/gkab1053 PMC8728234

[108] Leinonen R, Sugawara H, Shumway M, on behalf of the International Nucleotide Sequence Database Collaboration. The sequence read archive. Nucleic Acids Res. 2011;39:19–21. 10.1093/nar/gkq1019. 21062823 10.1093/nar/gkq1019 PMC3013647

[109] Kim D, Paggi JM, Park C, Bennett C, Salzberg SL. Graph-based genome alignment and genotyping with HISAT2 and HISAT-genotype. Nat Biotechnol. 2019;37:907–15. 10.1038/s41587-019-0201-4. 31375807 10.1038/s41587-019-0201-4 PMC7605509

[110] Li H, Handsaker B, Wysoker A, Fennell T, Ruan J, Homer N, et al. The sequence Alignment/Map format and samtools. Bioinformatics. 2009;25:2078–9. 10.1093/bioinformatics/btp352. 19505943 10.1093/bioinformatics/btp352 PMC2723002

[111] Rempel A, Choudhary N, Pucker B. KIPEs3: automatic annotation of biosynthesis pathways. Preprint. Bioinformatics; 2022. 10.1101/2022.06.30.498365. 10.1371/journal.pone.0294342 PMC10653506 37972102

[112] Ranwez V, Harispe S, Delsuc F, Douzery EJP. MACSE: multiple alignment of coding sequences accounting for frameshifts and stop codons. PLoS ONE. 2011;6:e22594. 10.1371/journal.pone.0022594. 21949676 10.1371/journal.pone.0022594 PMC3174933

[113] Price MN, Dehal PS, Arkin AP. FastTree: computing large minimum evolution trees with profiles instead of a distance matrix. Mol Biol Evol. 2009;26:1641–50. 10.1093/molbev/msp077. 19377059 10.1093/molbev/msp077 PMC2693737

[114] Letunic I, Bork P. Interactive tree of life (iTOL) v6: recent updates to the phylogenetic tree display and annotation tool. Nucleic Acids Res. 2024;52:W78–82. 10.1093/nar/gkae268. 38613393 10.1093/nar/gkae268 PMC11223838

[115] Bray NL, Pimentel H, Melsted P, Pachter L. Near-optimal probabilistic RNA-seq quantification. Nat Biotechnol. 2016;34:525–7. 10.1038/nbt.3519. 27043002 10.1038/nbt.3519

[116] Wickman H. ggplot2: elegant graphics for data analysis. New York: Springer-; 2016.

[117] Kuznetsova A, Brockhoff PB, Christensen RHB. LmerTest package: tests in linear mixed effects models. J Stat Softw. 2017;82:1–26. 10.18637/jss.v082.i13.

[118] Lenth RV. emmeans: Estimated Marginal Means, aka Least-Squares Means. 2025. https://rvlenth.github.io/emmeans/

[119] Searle SR, Milliken FMS. Population marginal means in the linear model: an alternative to least squares means. Am Stat. 1980;34:216–21. 10.1080/00031305.1980.10483031.

[120] R Core Team. R: A language and environment for statistical computing. 2024. https://www.R-project.org/

[121] Katoh K, Standley DM. MAFFT multiple sequence alignment software version 7: improvements in performance and usability. Mol Biol Evol. 2013;30:772–80. 10.1093/molbev/mst010. 23329690 10.1093/molbev/mst010 PMC3603318

[122] Waterhouse AM, Procter JB, Martin DMA, Clamp M, Barton GJ. Jalview version 2—a multiple sequence alignment editor and analysis workbench. Bioinformatics. 2009;25:1189–91. 10.1093/bioinformatics/btp033. 19151095 10.1093/bioinformatics/btp033 PMC2672624

[123] Meng EC, Goddard TD, Pettersen EF, Couch GS, Pearson ZJ, Morris JH, et al. UCSF chimerax: tools for structure Building and analysis. Protein Sci. 2023;32:e4792. 10.1002/pro.4792. 37774136 10.1002/pro.4792 PMC10588335

[124] NCBI. NCBI / SRA-ToolKit. 2025. https://github.com/ncbi/sra-tools

[125] Robinson JT, Thorvaldsdóttir H, Winckler W, Guttman M, Lander ES, Getz G, et al. Integr Genomics Viewer Nat Biotechnol. 2011;29:24–6. 10.1038/nbt.1754. 10.1038/nbt.1754 PMC3346182 21221095

[126] Quinlan AR, Hall IM. BEDTools: a flexible suite of utilities for comparing genomic features. Bioinformatics. 2010;26:841–2. 10.1093/bioinformatics/btq033. 20110278 10.1093/bioinformatics/btq033 PMC2832824

[127] Ou S, Su W, Liao Y, Chougule K, Agda JRA, Hellinga AJ, et al. Benchmarking transposable element annotation methods for creation of a streamlined, comprehensive pipeline. Genome Biol. 2019;20:275. 10.1186/s13059-019-1905-y. 31843001 10.1186/s13059-019-1905-y PMC6913007

[128] Tang H, Krishnakumar V, Zeng X, Xu Z, Taranto A, Lomas JS, et al. JCVI: A versatile toolkit for comparative genomics analysis. iMeta. 2024;3:e211. 10.1002/imt2.211. 39135687 10.1002/imt2.211 PMC11316928

[129] Altschul SF, Gish W, Miller W, Myers EW, Lipman DJ. Basic local alignment search tool. J Mol Biol. 1990;215:403–10. 2231712 10.1016/S0022-2836(05)80360-2

[130] Jaillon O, Aury J-M, Noel B, Policriti A, Clepet C, Casagrande A, et al. The grapevine genome sequence suggests ancestral hexaploidization in major angiosperm phyla. Nature. 2007;449:463–7. 10.1038/nature06148. 17721507 10.1038/nature06148

[131] Motamayor JC, Mockaitis K, Schmutz J, Haiminen N, Iii DL, Cornejo O, et al. The genome sequence of the most widely cultivated Cacao type and its use to identify candidate genes regulating pod color. Genome Biol. 2013;14:r53. 10.1186/gb-2013-14-6-r53. 23731509 10.1186/gb-2013-14-6-r53 PMC4053823

[132] Liu H-L, Harris AJ, Wang Z-F, Chen H-F, Li Z-A, Wei X. The genome of the paleogene relic tree Bretschneidera sinensis: insights into trade-offs in gene family evolution, demographic history, and adaptive SNPs. DNA Res. 2022;29:dsac003. 10.1093/dnares/dsac003. 35137004 10.1093/dnares/dsac003 PMC8825261

[133] Shyamli PS, Pradhan S, Panda M, Parida A. De Novo Whole-Genome assembly of Moringa Oleifera helps identify genes regulating drought stress tolerance. Front Plant Sci. 2021;12. 10.3389/fpls.2021.766999. 10.3389/fpls.2021.766999 PMC8712769 34970282

[134] Ming R, Hou S, Feng Y, Yu Q, Dionne-Laporte A, Saw JH, et al. The draft genome of the Transgenic tropical fruit tree Papaya (Carica Papaya Linnaeus). Nature. 2008;452:991–6. 10.1038/nature06856. 18432245 10.1038/nature06856 PMC2836516

[135] Yang R, Jarvis DJ, Chen H, Beilstein M, Grimwood J, Jenkins J, et al. The reference genome of the halophytic plant eutrema salsugineum. Front Plant Sci. 2013;4. 10.3389/fpls.2013.00046. 10.3389/fpls.2013.00046 PMC3604812 23518688

[136] Slotte T, Hazzouri KM, Ågren JA, Koenig D, Maumus F, Guo Y-L, et al. The capsella Rubella genome and the genomic consequences of rapid mating system evolution. Nat Genet. 2013;45:831–5. 10.1038/ng.2669. 23749190 10.1038/ng.2669

[137] Lee C-R, Wang B, Mojica JP, Mandáková T, Prasad KVSK, Goicoechea JL, et al. Young inversion with multiple linked QTLs under selection in a hybrid zone. Nat Ecol Evol. 2017;1:1–13. 10.1038/s41559-017-0119. 28812690 10.1038/s41559-017-0119 PMC5607633

[138] Krämer U, Weigel D, Felix B, Lara S, Phytozome. Sep. https://phytozome-next.jgi.doe.gov/. Accessed 12 2024.

[139] Cheng C-Y, Krishnakumar V, Chan AP, Thibaud-Nissen F, Schobel S, Town CD. Araport11: a complete reannotation of the Arabidopsis Thaliana reference genome. Plant J. 2017;89:789–804. 10.1111/tpj.13415. 27862469 10.1111/tpj.13415

[140] Sreedasyam A, Plott C, Hossain MS, Lovell JT, Grimwood J, Jenkins JW, et al. JGI plant gene atlas: an updateable transcriptome resource to improve functional gene descriptions across the plant Kingdom. Nucleic Acids Res. 2023;51:8383–401. 10.1093/nar/gkad616. 37526283 10.1093/nar/gkad616 PMC10484672

[141] Sayers EW, Bolton EE, Brister JR, Canese K, Chan J, Comeau DC, et al. Database resources of the National center for biotechnology information. Nucleic Acids Res. 2022;50:D20–6. 10.1093/nar/gkab1112. 34850941 10.1093/nar/gkab1112 PMC8728269

[142] Hoang NV, Sogbohossou EOD, Xiong W, Simpson CJC, Singh P, Walden N, et al. The gynandropsis Gynandra genome provides insights into whole-genome duplications and the evolution of C4 photosynthesis in cleomaceae. Plant Cell. 2023;35:1334–59. 10.1093/plcell/koad018. 36691724 10.1093/plcell/koad018 PMC10118270

[143] Fernandez-Pozo N, Metz T, Chandler JO, Gramzow L, Mérai Z, Maumus F, et al. Aethionema arabicum genome annotation using PacBio full-length transcripts provides a valuable resource for seed dormancy and brassicaceae evolution research. Plant J. 2021;106:275–93. 10.1111/tpj.15161. 33453123 10.1111/tpj.15161 PMC8641386

